# *De Novo* Single-Stranded RNA-Binding Peptides Discovered by Codon-Restricted mRNA Display

**DOI:** 10.1021/acs.biomac.3c01024

**Published:** 2023-12-05

**Authors:** Shota Nishikawa, Hidenori Watanabe, Naohiro Terasaka, Takayuki Katoh, Kosuke Fujishima

**Affiliations:** † Earth-Life Science Institute, Tokyo Institute of Technology, Ookayama, Meguro-ku, Tokyo 152-8550, Japan; ‡ School of Life Science and Technology, Tokyo Institute of Technology, Meguro-ku, Tokyo 152-8550, Japan; § Department of Chemistry, Graduate School of Science, The University of Tokyo, Bunkyo-ku, Tokyo 113-0033, Japan; ∥ Graduate School of Media and Governance, Keio University, Fujisawa 252-0882, Japan

## Abstract

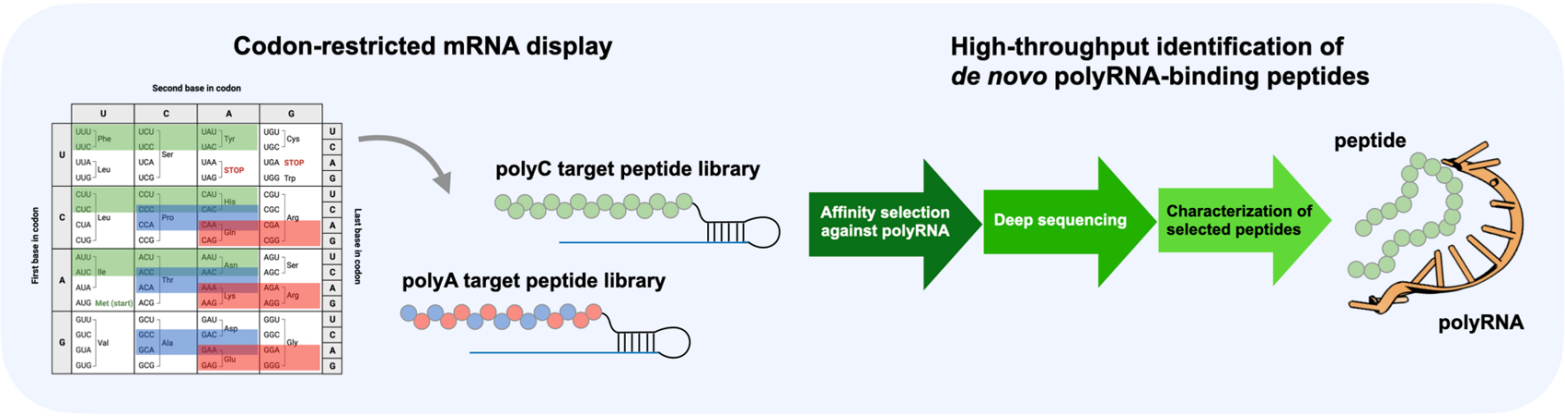

RNA-binding proteins participate in diverse cellular processes, including DNA repair, post-transcriptional modification, and cancer progression through their interactions with RNAs, making them attractive for biotechnological applications. While nature provides an array of naturally occurring RNA-binding proteins, developing *de novo* RNA-binding peptides remains challenging. In particular, tailoring peptides to target single-stranded RNA with low complexity is difficult due to the inherent structural flexibility of RNA molecules. Here, we developed a codon-restricted mRNA display and identified multiple *de novo* peptides from a peptide library that bind to poly(C) and poly(A) RNA with *K*_D_s ranging from micromolar to submicromolar concentrations. One of the newly identified peptides is capable of binding to the cytosine-rich sequences of the oncogenic Cdk6 3′UTR RNA and *MYU* lncRNA, with affinity comparable to that of the endogenous binding protein. Hence, we present a novel platform for discovering *de novo* single-stranded RNA-binding peptides that offer promising avenues for regulating RNA functions.

## Introduction

RNA-binding proteins (RBPs) are essential in core biological processes such as transcription, translation, gene expression, *etc*. More than 2000 RBPs have been reported and found to play critical roles in diverse cellular processes including DNA repair, post-transcriptional modification, and cancer progression through their interactions with RNAs.^1^ Moreover, RNA–protein interactions regulate viral replication and are often misregulated in neurological disorders and cancers.^2−5^ Therefore, RBPs have garnered considerable attention in both biological research and biotechnological applications.

RBPs commonly exhibit modular structures characterized by the presence of multiple repeats of a limited set of domains.^6^ The RNA-binding domains can assemble in diverse configurations, thereby creating adaptable RNA-binding surfaces. The modular architecture of the RBPs imparts the capacity to engage RNA with enhanced specificity and affinity compared to individual domains. Previously, significant advancements have been achieved in tailoring the RNA sequence specificity of Pumilio/fem-3-binding factor (PUF) repeat domains.^7,55^ A comprehensive recognition amino acid code for each of the four RNA bases has been determined, granting substantial flexibility in the design of protein specificity.^8^ Engineered PUF domains have been effectively integrated with diverse effector domains to serve various purposes, such as tracking RNA localization within cells,^9,10^ creating site-specific RNA endonucleases,^11^ and regulating the translation and stability of specific mRNA molecules *in vivo.*(12) These developments suggest a wide range of potential applications for RBPs as versatile tools for biotechnological applications. For example, peptides originating from RNA-binding domains such as RBM39, SAFA, or hnRNPK have been conjugated with the Penetratin cell-penetrating peptide (CPP) to effectively inhibit cancer cell proliferation by disrupting RNA–protein interactions.^13,14^

While nature has provided a repertoire of naturally occurring RBPs, these existing RBPs represent only a fraction of the potential repertoire of RBPs given a large fraction of the possible folds remains unexplored.^15^ RNA-binding domains that remain unexplored in nature not only hold promise for the expansion of our biotechnological toolkit but also harbor the potential to deepen our understanding of the fundamental mechanisms governing these molecular interactions. These profound insights, in turn, serve to facilitate the rational design of *de novo* RNA-binding proteins. Therefore, high-throughput screening for *de novo* RNA-binding peptides from a random peptide library offers a novel repertoire of RNA-binding peptides that have not existed in nature. Especially, targeting single-stranded RNA regions provides ground insights regarding nucleotide-specific recognition, which are highly beneficial for precise RNA probing and mRNA regulation. It is worth noting that single-stranded RNA regions play a crucial role in regulating virus infection and oncogene expression, emphasizing the importance of the development of *de novo* RNA-binding peptides.^16,17^ For example, cytosine-rich regions are present in the 5′ untranslated region of the RNA of hepatitis-C virus and poliovirus.^3,18^ The cytosine-rich regions regulate transcription levels *via* the interaction with the host poly(C)-binding proteins (PCBPs).^3,19,20^ Poly(A)-binding proteins (PABPs) are also known as essential eukaryotic mRNA regulators promoting the circularization of mRNA to enhance translation efficiency.^21−23^

*In vitro* display methods such as phage display, ribosome display, and mRNA display have been used as a powerful tool to screen novel peptide aptamers against versatile targets.^24−27^ Indeed, Li et al. recently reported a *de novo* hydrophobic-cationic RNA-binding peptide using phage display.^28^ mRNA display is a widely used *in vitro* selection technique to rapidly isolate functional peptides from libraries of more than 10^13^ molecules. It has been applied to directed evolution studies, drug development, and cellular proteomics analysis.^30,31^ mRNA display uses a stable covalent link between mRNA and its cognate polypeptide, thus enabling a selection for versatile protein functions such as protein binding, ATP-binding, and catalytic function including RNA ligation.^32−34^ mRNA display platform has also been diversified, for example, using a cell-free system to analyze protein–protein interaction and screening enzyme inhibitors.^35,36^ More recently, mRNA display combined with a reconstituted cell-free translation system (PURE mRNA display) has achieved high efficiency in *in vitro* selection by minimizing the carry-over of intrinsic cellular components.^29,37,38^ However, there is an apparent downside for the mRNA display when it comes to RNA binding. Especially if the target RNA contains a stretch of a single-stranded region (>5 nt), it can form a stable base-pairing between the mRNA, and therefore, mRNA harboring complementary sequences of the targeted RNA will be selected and hinder the selection process. To overcome this issue, Dr. Nemoto’s group invented the cDNA display method to mask mRNA with reverse-transcribed cDNA.^39,40^ They also applied this method with a codon-restricted library to screen for *de novo* tRNA (tRNA) binding peptides consisting of four prebiotically relevant amino acids.^41^ Similarly, Giacobelli et al. explored sequence variants of the C-terminal domain of ribosomal protein uL11 using PURE mRNA display and selected a primitive uL11 protein analog only composed of 10 prebiotically plausible amino acids.^42^ These previous studies demonstrate that peptides with a limited amino acid sequence can still interact with structurally conserved tRNA and rRNA. In this study, we mitigate the base-pairing problem by restricting the codon usage of the DNA library and demonstrate the efficiency of a codon-restricted mRNA display technique in targeting low-complexity single-stranded RNA (ssRNA), which can be further implemented to target biological RNA sequences.

## Materials and Methods

All oligos were ordered through FASMAC Inc. (Kanagawa, Japan). All peptides were chemically synthesized and purified with high purity (>90%) by GenScript (Piscataway, NJ) unless specified (methods for *in vitro* transcription; *in vitro* RNA transcription; puromycin-DNA-tag ligation; synthesis of mRNA-peptide conjugate by *in vitro* translation; gel purification; negative selection of mRNA-tag by the affinity with polyRNA; peptide selection based on the affinity with polyRNA; reverse transcription PCR (RT-PCR); sequence analysis; principal component analysis; calculation of hydrophobicity and net charge of peptide; enrichment analysis of combinatorial amino acid pairs; microscale thermophoresis are shown in the Supporting Information. The complete list of DNA and RNA oligos used in this work is available in Table S1).

### DNA Library Preparation

DNA libraries were prepared by annealing two single-stranded DNA (ssDNA) oligos and then extension *via* the Klenow fragment. The VMM/VRR codon library was produced from PolyAGCU-binding-F oligo (2 μM) and VMM/VRR codon library-R oligo (2 μM), whereas the HHY codon library was synthesized from PolyAGCU-binding-F oligo (2 μM) and HHY codon library-R oligo (2 μM). The oligo DNA pairs were mixed with dNTP (200 μM) in Klenow buffer (Takara Bio; Kusatsu, Shiga, Japan) and annealed by heating at 90 °C for 1 min and then cooling slowly to room temperature. Subsequently, 4 U of Klenow fragment (Takara Bio; Kusatsu, Shiga, Japan) was added to the reaction mixture. The primer was extended at 25 °C for 5 min and then 37 °C for 1 h followed by inactivation of the enzyme at 50 °C for 15 min. The linearized DNA libraries, VMM/VRR codon library, and HHY codon library were then purified with NucleoSpin Gel and PCR Clean-up Kit (Macherey-Nagel; Düren, Nordrhein-Westfalen, Germany). The DNA library was quantified by agarose gel electrophoresis (2.5% w/v agarose gel in 1× tris-acetate-EDTA (TAE)). The electrophoresis was run at 100 V for 40 min. The gel was then stained with SYBR Gold Nucleic Acid Gel Stain (Invitrogen; Waltham, MA). Gel images were captured and analyzed using an Amersham Imager 680 (GE Healthcare; Chicago, IL). Nucleic acid concentrations were quantified with a NanoDrop2000c spectrophotometer (Thermo Fisher Scientific; Waltham, MA).

### Codon-Restricted mRNA Display Method

We followed the basic protocols of the PURE mRNA display method and made minor updates for this study.^38^ For details, see the Supporting Methods. In brief, RNA libraries were produced by *in vitro* transcription from a DNA library, followed by puromycin-DNA-tag ligation. The ligated products are separated by gel electrophoresis and purified from the gel with electroelution and ethanol precipitation. As a preliminary step, an undesired mRNA-tag with an affinity to polyRNA is removed from the mRNA-tag pool. The remaining mRNA-tag is then used for *in vitro* translation using a cell-free system. Peptide-mRNA conjugates were gel-purified and mixed with polyRNA displayed on strep-tactin-immobilized magnetic beads. Biotin-eluted samples were amplified by reverse transcription PCR to obtain a DNA library for the following selection round. The above procedures are repeated for the next round of selection.

### Next-Generation Sequencing

Next-generation sequencing (NGS) was applied to the DNA library from each round (round 0 to round 7) using a MiSeq system (Illumina; San Diego, CA). A Qubit 2.0 fluorometer (Invitrogen; Waltham, MA) with a Qubit dsDNA HS assay kit (Thermo Fisher Scientific; Waltham, MA) was used to quantify sequencing libraries precisely. DNA sequencing libraries were generated using a NEBNext Ultra II DNA Library Prep kit (New England Biolabs, Inc.; Ipswich, MA). To enable multiplex analysis, unique index sequences were added to the DNA sequencing library by NEBNext Multiplex Oligos for Illumina (Index Primers Sets 1 and 2; New England Biolabs, Inc.; Ipswich, MA). Finally, the lengths of the constructed DNA libraries were confirmed by an Agilent 2100 Bioanalyzer (Agilent Technologies; Santa Clara, CA), and paired-end sequencing was performed using a Miseq Reagent Kit v3 (600 cycles).

### Spectral Shift Experiment

Chemically synthesized Cyanine5 (Cy5)-labeled oligonucleotides were obtained from FASMAC Inc. (Kanagawa, Japan). While AP1 and AP2 were completely dissolved in the binding buffer (tris-HCl (10 mM; pH 8.0) and Tween 20 (0.05%, v/v)), both CP1 and CP2 formed aggregates when the concentration reached above 10 μM. Therefore, aggregation-free SpS measurement was applied to determine the dissociation constant (*K*_D_). Spectral shift experiment (SpS) was conducted with the identified RNA-binding peptides in three independent experimental trials on a Monolith X (NanoTemper Technologies; Munich, Bayern, Germany).^43^ The oligonucleotide solutions were prepared in a buffer containing tris-HCl (20 mM; pH 8.0) and Tween 20 (0.1%, v/v), and the peptide solutions were prepared in RNase-free water. A 2-fold dilution series of the unlabeled peptides was prepared in 20 nM oligonucleotide, with the final concentrations of peptides ranging from 10 μM to 0.3 nM or 500 to 15 nM. Samples were incubated for 30 min at room temperature. Following incubation, the samples were filled into standard treated capillaries (NanoTemper Technologies; Munich, Bayern, Germany) and subsequently subjected to SpS detection analysis. General settings were applied for all SpS experiments as follows: manual temperature control: 25 °C; LED laser: red. LED power settings were chosen individually for each sample by adjusting the LED power to yield a fluorescence signal of at least 200 units. For SpS detection, the fluorescence was recorded simultaneously at 650 and 670 nm for 5 s without the application of a temperature change. The obtained values were normalized and plotted against the peptide concentration. The dissociation constant was then determined by using a single-site model to fit the curve.

### Circular Dichroism Spectroscopy

The circular dichroism (CD) spectra were recorded using a J-1100 CD spectrophotometer (JASCO; Hachioji, Tokyo, Japan) over the wavelength range of 190–250 nm in steps of 1 nm with an averaging time of 1 s per step. Peptides were prepared in a buffer containing tris-HCl (pH 8.0; 10 mM) and Tween 20 (0.05%, v/v) with a final concentration of 0.2 mg/mL, and the CD spectra were measured in 1 mm path-length quartz cells at 25 °C. The CD signal was obtained as ellipticity in units of millidegrees, and the resulting spectra were averaged from two scans and the buffer spectrum was subtracted. All CD measurements were performed twice.

## Results

### Peptide Library Design

**Figure 1:**
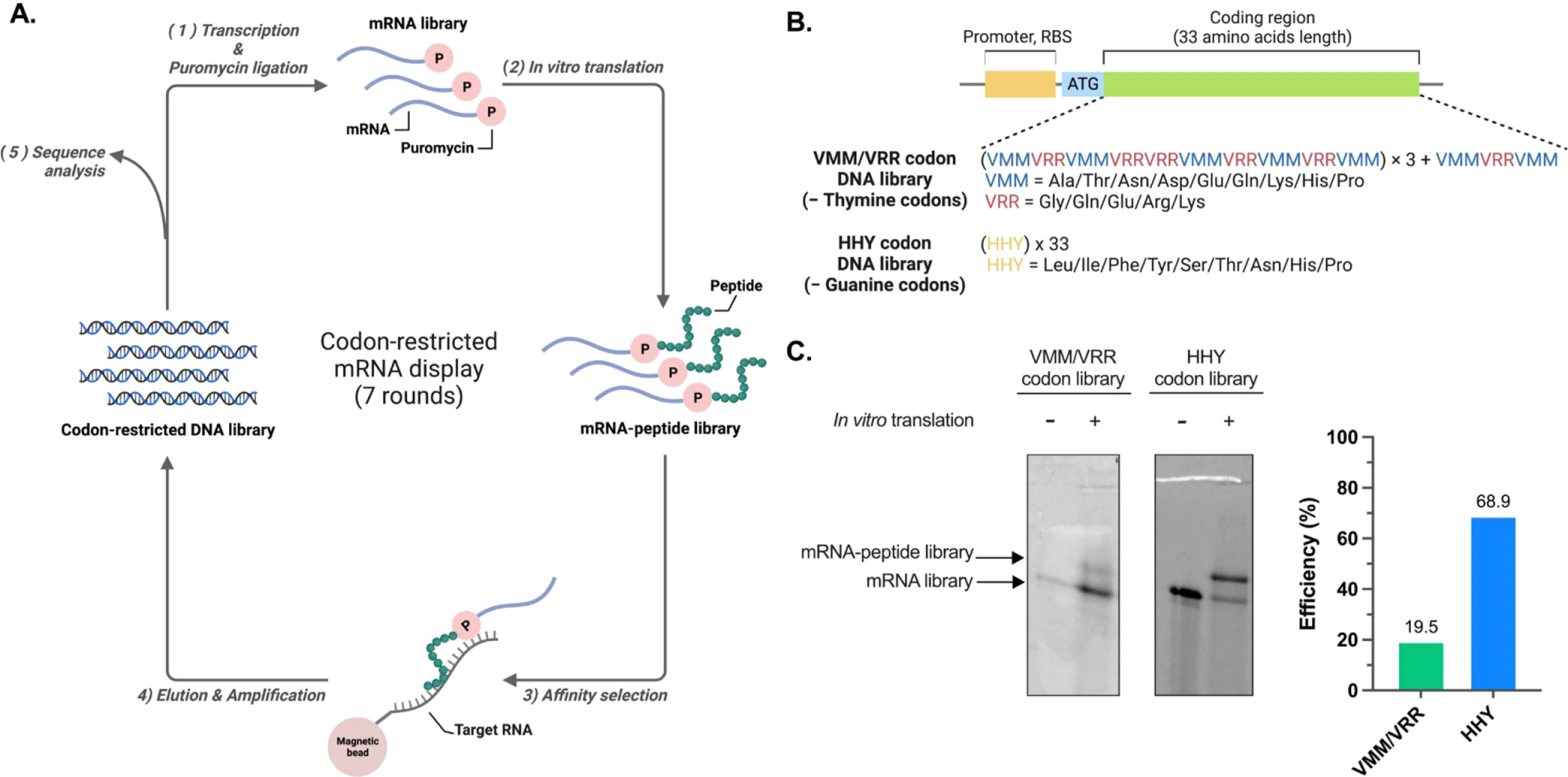
Selection of *de novo* peptide libraries against single-stranded RNA using codon-restricted mRNA display. (A) Schematic representation of codon-restricted mRNA display. The selection was conducted seven times in total based on the affinity with either poly(A) or poly(C) RNA. The DNA libraries from each round (rounds 0–7) were subjected to next-generation sequencing (see detailed schematics in Figure S1). (B) *In vitro* mRNA display enables the creation of peptide libraries from codon-restricted DNA libraries. The coding region of the VMM/VRR codon DNA library consists of “VMM” or “VRR” degenerate codons, while the HHY codon library comprises “HHY” degenerate codons (the codon translation table is available in Figure S2). (C) Synthesis of mRNA-peptide conjugates *via in vitro* translation. *In vitro* translation reactions were conducted using puromycin-DNA-tagged mRNA as templates and separated on SDS-polyacrylamide gels (stacking gel: 3.5% w/v polyacrylamide gel; separation gel: 10% w/v polyacrylamide gel). The image was captured by using fluorescein isothiocyanate (FITC) fluorescence (left). The gel images taken in each round are available in Figure S4. The efficiency of the conjugate formation was calculated based on the band intensities of the conjugate and free mRNA-tag (right).

The scheme for codon-restricted mRNA display is outlined in Figure 1A. We designed two types of DNA libraries encoding 33-mer randomized polypeptide sequences, each targeting 12-mer poly(A) and poly(C) RNA (Figure 1B). To avoid unwanted base-pairing between mRNA and the target RNA, codons including the complementary nucleotide were eliminated from the coding sequence. For example, the DNA library targeting poly(A) RNA comprises two types of degenerate “VMM” and “VRR” codons both lacking thymine nucleotide, which encodes a total of nine (Ala/Thr/Asn/Asp/Glu/Gln/Lys/His/Pro) and five (Gly/Gln/Glu/Arg/Lys) types of amino acids, respectively (Figure S2A), whereas the DNA library targeting poly(C) RNA comprises a single degenerate codon “HHY” lacking guanine nucleotide, which encodes a total of nine types of amino acids (Leu/Ile/Phe/Tyr/Ser/Thr/Asn/His/Pro) (Figure S2B).

### *In Vitro* Selection of *De Novo* RNA-Binding Peptides

The two DNA libraries were generated (Figure S3A) and subjected to our codon-restricted mRNA display method. A puromycin-DNA tag was attached to the 3′ ends of the mRNA library created *in vitro* (Figure S3BC). Next, through *in vitro* translation, we isolated the mRNA-peptide conjugates (Figure 1C). The conjugate formation efficiency of the HHY codon library reached 68.9%, in contrast to the VMM/VRR codon library, which was only 19.5%. This trend was consistent throughout multiple rounds of selection (Figure S4). The mRNA-peptide conjugates were subjected to affinity selection against poly(A) or poly(C) RNA. Finally, to generate a cDNA library for the next round of selection and deep sequencing, the eluted molecules were subjected to reverse transcription PCR (RT-PCR) (Figure S5). Seven rounds of selection were performed, with the cDNA sequences obtained from each round analyzed by the Illumina Miseq platform to monitor the enrichment of specific amino acids or peptide motifs expected to play a role in RNA binding.

### Sequence Analysis

**Figure 2:**
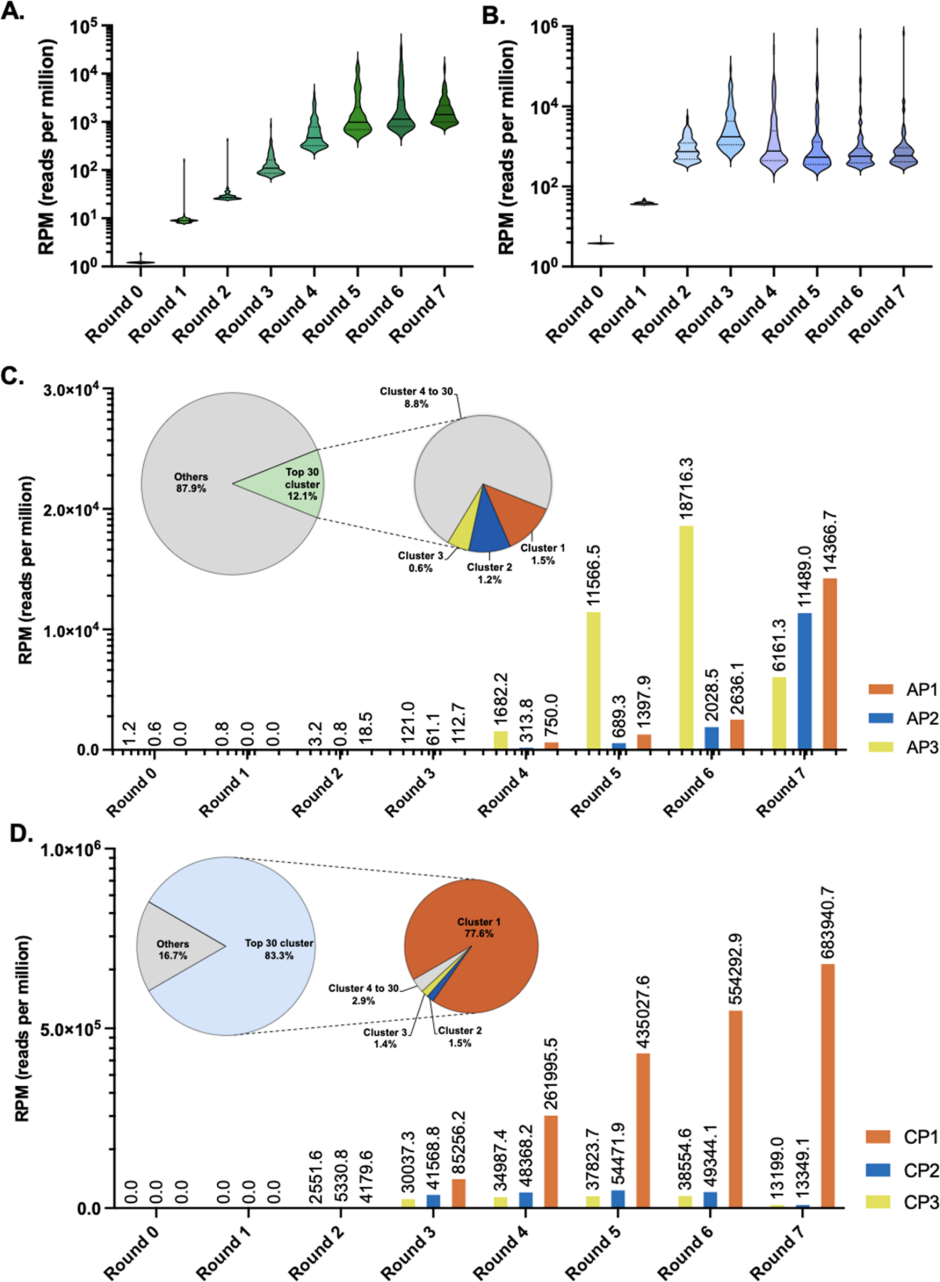
Sequence analysis of RNA-binding peptides. (A, B) Violin plots displaying reads per million (RPM) values for the top 100 enriched peptide sequences per selection round. The dashed lines indicate quartile values, while the solid line represents the mean. (C, D) Pie charts illustrating the proportions of the top 30 enriched peptide clusters from round 7 of screening. The RPM values of the top sequences within each cluster are depicted across the selection rounds: (C) poly(A) RNA-targeting VMM/VRR codon library and (D) poly(C) RNA-targeting HHY codon library. AP1–3 and CP1–3 are the top sequences of the three most enriched clusters in round 7.

The peptide sequences obtained *via* deep sequencing were analyzed by FastAptamer software to count and rank the unique peptide sequences based on the normalized reads per million (RPM) values.^44^ We first analyzed the top 100 enriched peptides from each round to compare the progression of the enrichment throughout the selection. For the VMM/VRR codon library, the average RPM values of the top 100 enriched peptides constantly increased until round 6 (Figure 2A). In contrast, the HHY codon library has reached the RPM peak at round 3 and gradually decreases subsequently (Figure 2B). We further performed clustering analysis with the peptide sequences in round 7 from each library based on their sequence similarity. For the VMM/VRR codon library, the 30 most enriched sequence clusters account for only 12.1% of all peptide sequences, with the most enriched cluster sequences sharing 1.5% of all peptide sequences (Figure 2C). In contrast, the HHY codon library was dominated by a single peptide cluster sequence covering 77.6% of all sequences, outcompeting the second-ranked cluster peptide sequences (Figure 2D).

### Amino Acid Composition Analysis of Enriched Peptide Sequences

**Figure 3:**
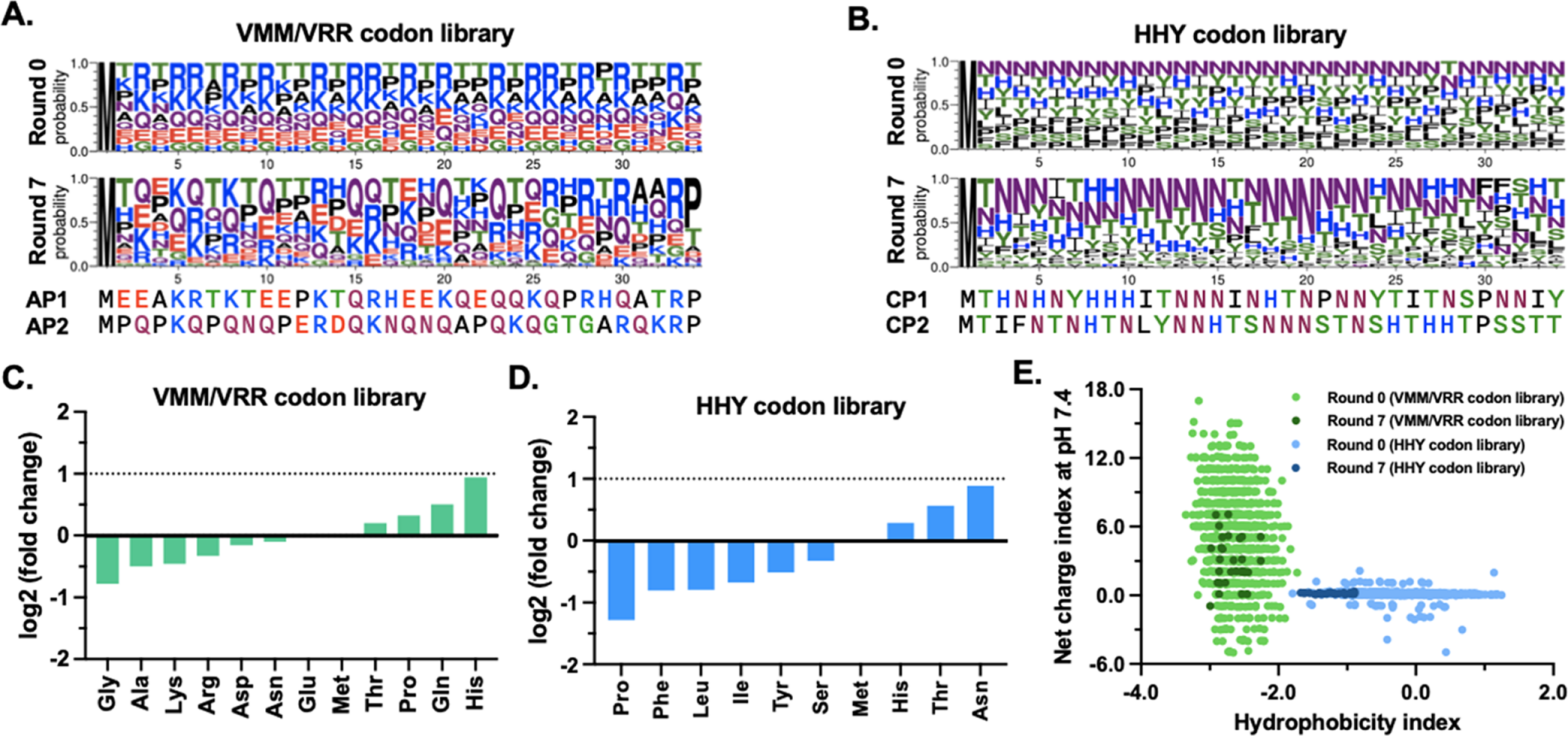
Amino acid composition feature of selected RNA-binding peptides. (A, B) Sequence logos were generated from peptide sequences in round 0 and round 7, respectively. For round 0 (before screening), 1000 randomly selected peptide sequences were used. The top sequences of the two most enriched clusters are listed below: (A) VMM/VRR codon library and (B) HYY codon library. (C, D) Log2-fold changes of amino acid frequencies after the whole selection are shown. The dotted line indicates a log2-fold change threshold of 1.0: (C) VMM/VRR codon library and (D) HHY codon library. (E) The two-dimensional plot represents hydrophobicity vs net charge indexes of individual peptides from round 0 (randomly selected 1000 sequences) and round 7 (top 30 sequences) libraries.

To further analyze the sequence profile of our enriched peptides, the consensus sequence logos were created from the top sequences of the 30 most enriched 30 clusters (Tables S2 and S3). As a result, in the poly(A) RNA-targeting VMM/VRR codon library, we observed a clear enrichment of glutamine (Q) appearing as the most frequently used amino acid at several positions (Figure 3A). Overall enrichment of asparagine (N) followed by threonine (T) and histidine (H) was observed throughout the poly(C) RNA-targeting HHY codon library (Figure 3B). Next, we calculated the log2-fold change in amino acid frequencies after seven rounds of selection. For the VMM/VRR codon library, the frequency of the positively charged amino acids Lys (K) and Arg (R), which are known to be common at RNA-binding interfaces, decreased (log2-fold change = −0.46 and −0.33, respectively), while the frequency of the hydrophilic amino acids His (H), Gln (Q), Pro (P), and Thr (T) increased (log2-fold change = 0.94, 0.50, 0.32, and 0.20, respectively) (Figure 3C). Similarly, in the HHY codon library, the frequency of hydrophilic amino acids Asn (N), Thr (T), and His (H) increased (log2-fold change = 0.89, 0.56, and 0.29, respectively) and hydrophobic/aromatic amino acids Ile (I), Leu (L), Tyr (Y), and Phe (F) decreased (log2-fold change = −0.68, −0.79, −0.51, and −0.81, respectively) (Figure 3D). The overall trend in the amino acid frequency change is also visualized as a distribution of the principal component analysis plot (Figure S6). We observed a significant enrichment of the amino acids harboring the carboxamide group in both libraries (Gln (Q) in the VMM/VRR codon library and Asn (N) in the HHY codon library). Also, the two-dimensional plot of net charge vs hydrophobicity index has shown that the enriched peptides from the HHY codon library remained electrically neutral (at pH 7.4) while significantly shifting toward lower hydrophobicity within the peptide sequence space (Figure 3E). Whereas the peptides from the initial VMM/VRR codon library possessed diverse net charge profiles (−5 to 15) along with overall low hydrophobicity profiles (−2 to −3), enriched peptides converged toward a narrow net neutral to positive charge window (0–5). The enrichment toward low hydrophobicity indicates the overall soluble feature of the selected peptide pool.

### Dissociation Constant Analysis of the Identified RNA-Binding Peptides

**Figure 4:**
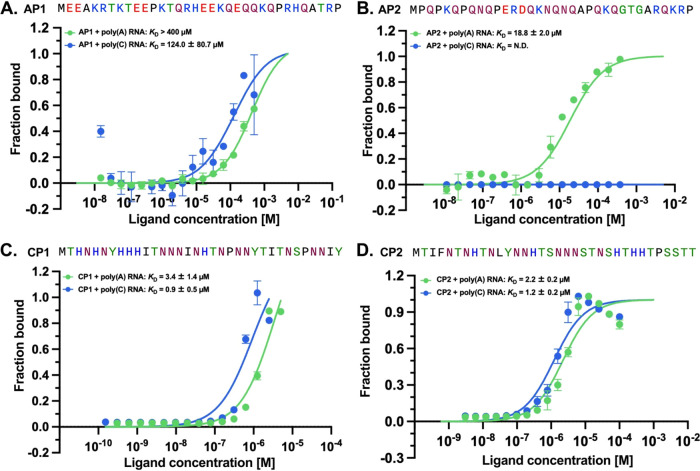
Thermodynamic plots of the identified RNA-binding peptides against polyRNAs. Thermodynamic plots were generated to determine *K*_D_ values between the identified peptides and polyRNAs. The *K*_D_ values were determined by spectral shift (SpS) experiments for (A) AP1, (B) AP2, (C) CP1, and (D) CP2. The bars represent the mean ± SE from three independent experimental replicates.

We chose the top two most enriched sequence clusters from each library (CP1/CP2 and AP1/AP2) to test their affinity against polyRNAs. As a result, AP1 only expressed very low affinity toward poly(A) RNA (Figure 4A), whereas the three peptides AP2, CP1, and CP2 have exhibited apparent affinity to their targeted polyRNAs with the dissociation constants (*K*_D_) of 18.8, 0.9, and 1.2 μM, respectively (Figure 4B–D). CP1 and CP2 exhibited slightly high affinity with the target poly(C) RNA over the nontarget poly(A) RNA, although they retain the affinity with poly(A) RNA within a range of single μM (*K*_D_) (Figure 4C,D). On the contrary, AP2 revealed 1 to 2 orders of magnitude lower binding affinity toward its target RNA than CP1 and CP2; however, AP2 exhibited specific interaction with poly(A) RNA (Figure 4B).

Further, by using CP1 and AP2 as a model for *de novo* RNA-binding peptides, we tested the sugar recognition by switching the target sequence from polyRNA to polyDNA. As a result, both AP2 and CP1 still exhibited affinity toward their DNA targets while AP2 but with approximately 2-fold less affinity compared to that of RNA (Figure S7). Then, to examine the role of nucleobase recognition in RNA–peptide interactions, we performed additional affinity analysis using the RNA hybridized with antisense DNA. Our findings revealed that AP2 lost its affinity for hybridized poly(A) RNA (Figure S8A). Similarly, CP1 exhibited a significantly lower affinity for poly(C) RNA, with approximately a 6-fold decrease compared to its affinity for nonhybridized poly(C) RNA (Figure S8B). This observation strongly suggests that nucleobase–peptide interactions play a significant role in determining the affinity.

### Role of Enriched Dipeptide Motifs Found in the RNA-Binding Peptide Sequences

**Figure 5:**
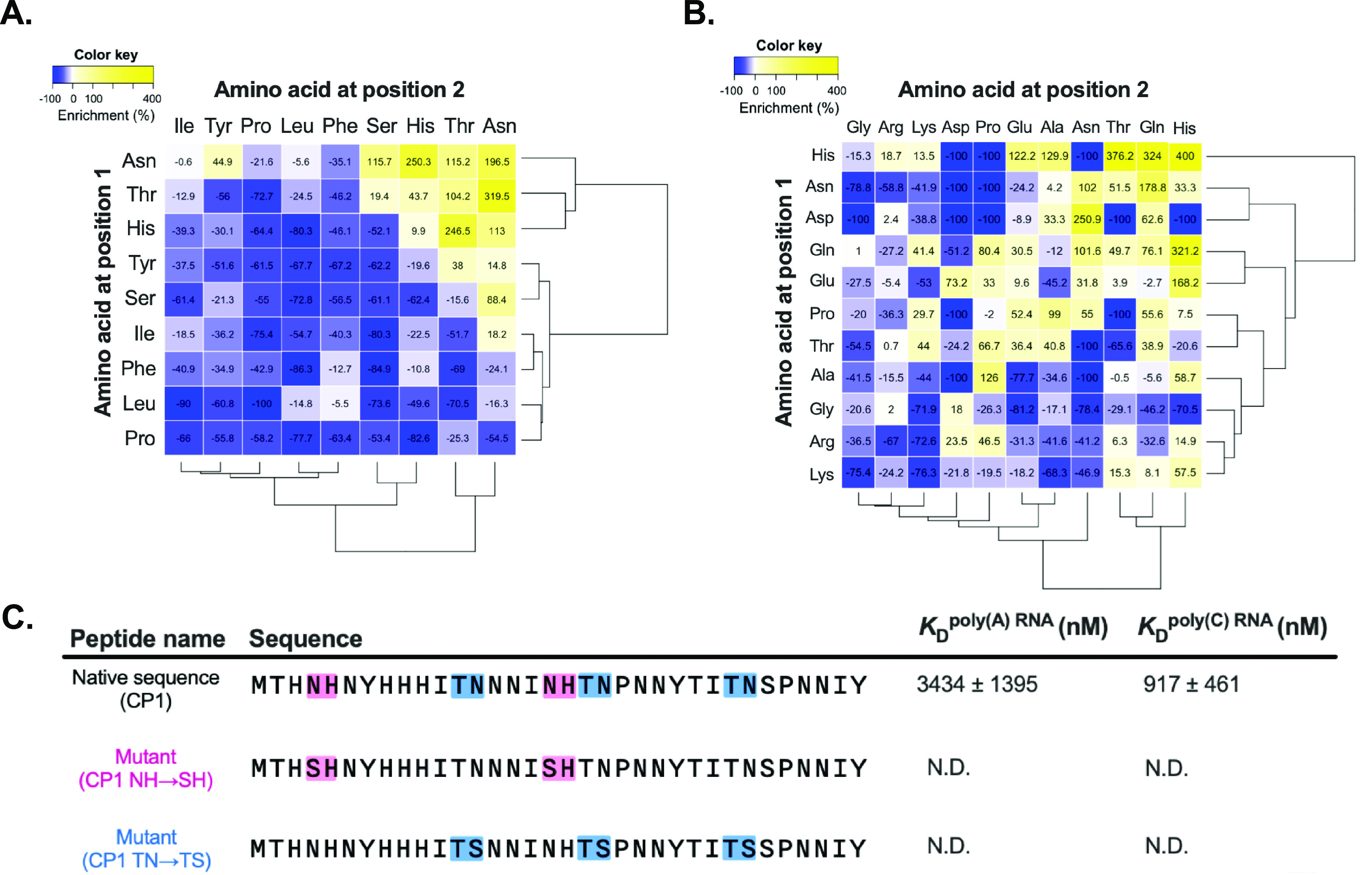
Identification of the enriched combinatorial amino acid pairs and dissociation constant analysis of the identified RNA-binding peptide variants. The enrichment scores of combinatorial amino acid pairs were calculated by comparing the frequency between the peptides from rounds 0 and 7 of each library. Hierarchical clustering was employed to visualize the enrichment scores through heat mapping for (A) the HHY codon library and (B) the VMM/VRR codon library. (C) Table showing the dissociation constants between the peptide variants and polyRNAs. NH-to-SH substitution (pink) and TN-to-TS substitution (blue) are highlighted. The complete thermodynamic plots are available in Figure S10.

Next, we calculated the frequency of combinatorial amino acid pairs (i.e., dipeptide motifs) enriched in the two libraries, expecting overrepresented dipeptide motifs to play an essential role in RNA binding. We found that the top 100 selected poly(C) RNA-binding peptides were strictly enriched in the dipeptide motifs comprising the (His/Asn/Thr) amino acid combinations (Figure 5A), whereas the top 100 selected poly(A) RNA-binding peptides still comprise a broader spectrum of the enriched dipeptide motifs that include one of the six (His/Asn/Gln/Glu/Pro/Thr) amino acids (Figure 5B).

To experimentally validate the function of enriched dipeptide motif residues, we substituted QNQ with TTT in the AP2 peptide due to the significant enrichment of QN and NQ motifs, which both contain glutamine, which is highly enriched in the VMM/VRR codon library. Nevertheless, the *K*_D_ and specificity toward adenine were unaffected by the replacement of QNQ with TTT (Figure S9A). Additionally, we created a truncated peptide called “AP2-short”, consisting of 6–26 AP2 residues, to identify the specific residues responsible for adenine recognition. As a result, the affinity of AP2-short was approximately 5 times lower (*K*_D_: 82.0 μM) than that of full-length AP2. However, the truncated peptide still recognized poly(A) RNA specifically (Figure S9B). Finally, we introduced a single point mutation, replacing glutamine (Q) with serine (S), in the middle of the sequence within AP2-short. This mutation resulted in a decreased dissociation constant (*K*_D_: 114.7 μM), demonstrating that a single residue substitution can increase the overall affinity toward poly(A) RNA. The truncated region (1–5 and 27–33 positions) contains two of the most enriched amino acids in the top 30 clusters of the VMM/VRR library: glutamine (Q) and proline (P), as well as the two basic amino acids, lysine (K) and arginine (R), which are well-known for their interaction with RNA and DNA. Additionally, five enriched dipeptide motifs (PQ/QP/PK/QK/RP) are found in the truncated region and could potentially serve as RNA-binding interfaces. However, additional mutagenesis is necessary to confirm the involvement of specific residues in the interaction between RNA and peptide.

**Figure 6:**
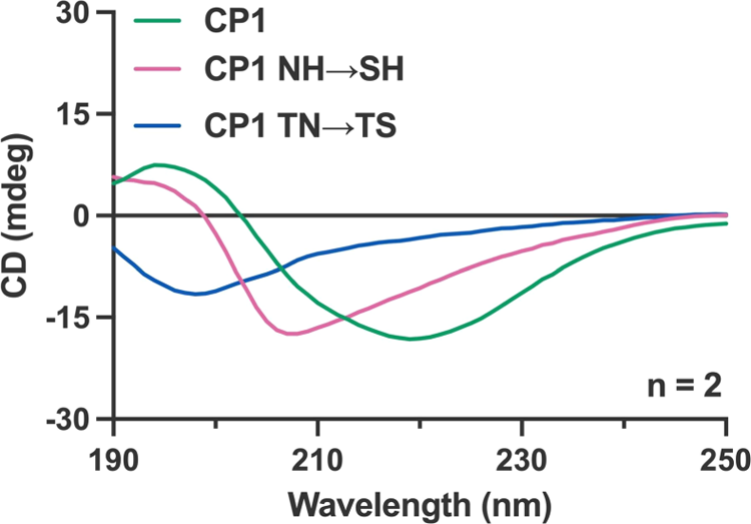
CD spectra of the CP1 peptide and its variants. CD spectra were measured for each peptide with a concentration of 0.2 mg/mL in a buffer containing tris-HCl (pH 8.0; 10 mM) and Tween 20 (0.05%, v/v). The CD spectra were acquired in duplicate and averaged: CP1 (green), CP1 NH → SH (pink), and CP1 TN → TS (blue).

As for the CP1 peptide, we designed two variants by replacing Asn with Ser within the two most enriched dipeptide motifs (NH and TN) to determine the residues responsible for the RNA-binding capability. Serine mutation was used instead of the commonly used alanine to avoid decreasing the peptide solubility. Both mutants, NH to SH and TN to TS, lost the binding capability against polyRNAs to below detection limits (Figures 5C and S10), indicating that asparagine alongside histidine and threonine residues are involved in the recognition of polyRNAs. In an attempt to reveal the effect of the mutations on the peptide structure, CD spectroscopy was performed with native CP1 and its mutant peptides (Figure 6). The CD spectrum of CP1 showed a distinct maximum at 195 nm and a minimum at 218 nm, indicating the formation of a β-sheet structure. On the other hand, the TN-to-TS mutant peptide displayed a distinct random coil structure, with a single minimum at 198 nm, indicating that TN-to-TS mutagenesis induced a structural transition from a β-sheet into a random coil structure. Likewise, NH-to-SH mutagenesis resulted in a shift of the minimum from 218 nm in the native peptide to 208 nm, indicating a partial loss in the β-sheet structure to form a random coil structure. Altogether, the mutagenesis resulted in the structural transition from β-sheet toward the random coil signal, emphasizing the importance of β-sheet structure for the RNA-binding capability of CP1.

Finally, to observe the effect of TN and NH motifs, the AP2 peptide sequence was modified by replacing the two QNQ motifs with the TNH motif (combination of TN and NH). This modification aimed to enhance AP2’s poly(C) RNA-binding capability while retaining its inherent ability to bind poly(A) RNA. By replacing QNQ with TNH, AP2 recognized poly(C) RNA as expected (*K*_D_: 120.3 μM) and displayed notably higher affinity for poly(A) RNA (Figure S9C). This observation is consistent with CP1’s proficiency at binding poly(A) RNA. Thus, the TNH sequence acts as a versatile ssRNA-binding motif that prefers cytosine.

### Synthetic Poly(C) RNA-Binding Peptide Interacts with Cytosine-Rich Oncogenic RNA Motifs in a Human Plasma-like Medium

**Figure 7:**
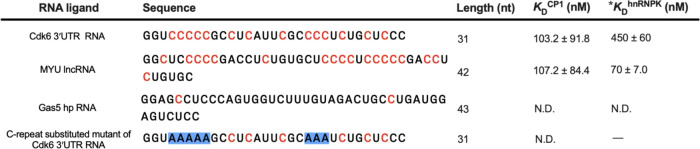
Assessment of the affinity of poly(C) RNA-binding peptides to cytosine-rich oncogenic RNA motifs. The table displays the dissociation constant (*K*_D_^CP1^) between hnRNPK-associated RNAs and CP1 peptides. The *K*_D_^CP1^s were measured using microscale thermophoresis in a human plasma-like medium. Cytosines predicted to be single-stranded within RNA ligands are highlighted in red. Cytosine-to-adenine substitution is highlighted in blue. The predicted secondary structures are available in Figure S11. The complete thermodynamic plots are shown in Figure S12. *The *K*_D_^hnRNPK^s are the dissociation constants between hnRNPK-associated RNAs and hnRNPK determined by fluorescence anisotropy binding assay reported in the previous study.^45^

To evaluate the synthetic ssRNA-binding peptide’s affinity for biologically derived RNA targets, we have chosen two cytosine-rich RNA motifs from the oncogenic Cdk6 3′UTR RNA and *MYU* lncRNA. These motifs have previously been identified as targets of hnRNPK,^45^ and they both consist of extended cytosine repeats (>4 nt) within a necessary single-stranded region for the progression of colon cancer cells. We found that the CP1 peptide can bind to RNA motifs with submicromolar *K*_D_s. Specifically, it can bind to both Cdk6 3′UTR RNA (103.2 nM) and *MYU* lncRNA (107.2 nM) in a medium that mimics human plasma (see Figure 7). Notably, CP1 showed a comparable *K*_D_ to that of hnRNPK with Cdk6 3′UTR RNA (*K*_D_: 450 nM).^45^ On the contrary, CP1 did not demonstrate any affinity toward the established negative control for hnRNPK cytosine-poor Gas5 hp RNA, suggesting a preference for a single-stranded cytosine repeat. As CP1 exhibited a stronger inclination toward Cdk6 3′UTR RNA compared to hnRNPK, we proceeded to mutate the cytosine-rich, single-stranded regions of Cdk6 3′UTR RNA to confirm the contribution of these polyC regions to CP1 binding. The replacement of two extended cytosine repeats positioned at 4–8 and 20–22 with adenine repeats (C-repeat-substituted mutant of Cdk6 3′UTR RNA) resulted in losing the binding ability (Figure 7), demonstrating that CP1 interacts with the polyC regions of Cdk6 3′UTR RNA.

## Discussion

mRNA and cDNA display has been commonly used as efficient tools to explore large peptide sequence space against versatile binding targets (library size can reach up to ∼10^13^ molecules). Previous *in vitro* selection studies have shown that various RNA- and DNA-binding peptides can be selected.^41,42,46,47^ However, screening for *de novo* peptides that can interact with low-complexity ssRNA remains a major challenge using the mRNA display due to unwanted base-pairing between mRNA and target RNA. Therefore, we decided to take a new approach to combine a codon-restricted DNA library with mRNA display to overcome the problem mentioned above.

Our *in vitro* selection successfully led to the identification of four novel *de novo* RNA-binding peptides (AP1/AP2/CP1/CP2) interacting with poly(A) or poly(C) RNA. Deep sequencing revealed significant glutamine (Q) and asparagine (N) composition enhancement in the top 30 enriched cluster sequences of each library. It has been known that Watson–Crick and Hoogsteen-type interactions can occur between the Q/N carboxamide group and purine/pyrimidine nucleobases (Figure S13).^48,49^ It was, therefore, not surprising that all four newly identified RNA-binding peptides are enriched in Q/N residues (AP1: 18%, AP2: 39%, CP1: 36%, CP2: 27%). The most significant case was the CP1 peptide, relying solely on the asparagine residues within the top 2 most enriched TN and NH dipeptide motifs (enrichment >250%; Figure 4B). The fact that CP1 binds to poly(C) RNA with a *K*_D_ of 900 nM, which is approximately 4- and 7-fold stronger compared to binding poly(A) RNA and poly(C) DNA, suggests that the asparagine carboxamide group is in contact with both bases (A and C) as well as 2′ OH of the ribose. This is consistent with the evidence that the carboxamide group can form a hydrogen bond with 2′ OH either directly or indirectly bridged by a water molecule.^49,50^ The decrease in *K*_D_ was not as prominent in the AP2-poly(A) DNA interaction, suggesting that 2′ OH is less important for AP2 recognition. Indeed, AP2 affinity toward poly(A) RNA (*K*_D_: 18.8 μM) was orders of magnitude lower than that of CP1, and specificity toward adenine moiety may suggest the recognition *via* the Hoogsteen pseudo-pair. At least in the case of DNA duplex, Hoogsteen base-pairing is ∼3 kcal/mol higher in energy than the Watson–Crick base-pairing,^51^ which may explain the relatively low affinity of AP2 and AP1. Because the configuration of the cytosine–asparagine interaction in the HHY library is limited to the Watson–Crick pseudo-pair (Figure S13), we speculate that limited structural configuration along with the lack of positively charged amino acids likely resulted in the steep and noncontinuous fitness landscape leading to the dominance of CP1 cluster sequences occupying 77.6% of the entire population after seven rounds of selection.

As opposed to the HHY library, the variety of amino acids that can interact with polynucleotides is clearly diverse in the VMM/VRR library. We observed that the two QNQ sequences consisting of the highly enriched QN/NQ dipeptide motifs (enrichment >100%; Figure S9A) can be replaced by TTT sequences, suggesting either (i) the hydroxyl group in threonine can complement the hydrogen bonding of the carboxamide group, (ii) peptide backbone is involved in the interaction, or (iii) these residues are not in direct contact with the poly(A) RNA. While truncation and point mutation led to a stepwise decrease of AP2 affinity but did not entirely lose the binding, we consider the involvement of multiple amino acid residues that contain other enriched dipeptide motifs. Also, since we did not incorporate metal cations during the selection, the previously reported cation-mediated interaction between acidic amino acids and RNA^42^ is unlikely to be involved. Therefore, we assume that net negative acidic peptides (net charge < 0) were unfavorable under our RNA-binding condition, resulting in a constrained chemical parameter space of the enriched peptides after seven rounds of selection. A clear trend toward decreasing hydrophobicity in both libraries may indicate that the selected peptides do not prefer forming a tertiary soluble structure with hydrophobic core, in order to maximize the interface with the unstructured linear RNA. The fact that RNA-binding capacity was affected by a single amino acid (Q or N) substitution, as well as N- and C-terminal end truncation, further supports this hypothesis.

In nature, structured cellular PCBPs possess multiple KH domains that bind to cytosine triplet at the hydrophobic cleft formed by a small GXXG loop and variable loop.^52^ The positively charged amino acid residues surrounding the cleft attract the oligonucleotide, and specific recognition of cytosine is realized by a dense network of hydrogen bonding with conserved amino acid side chains (Asp, Arg, Glu, Gly, Ile, and Lys).^53^ Such a coordination requires a stable tertiary structure that short peptides cannot provide. However, our novel poly(C) RNA-binding peptide (CP1), which is enriched with Asn, Thr, and His, can interact with RNA that is rich in cytosine with a comparable affinity to that of hnRNPK with the KH domain. This suggests that the RNA-binding mechanism differs significantly from that of typical RNA-binding domains. Since the CP1 peptide contains four motifs (NH and TN) that recognize cytosine within its sequence, it is possible that having multiple cytosine-recognizing motifs contributes to the preference for cytosine repeats. Additionally, secondary structure analysis performed through CD spectroscopy confirmed a distinct β-sheet structure in CP1, whereas the two unbound variant peptides led to complete/partial disruption of this β-sheet structure (Figure 6). This result indicated that unlike the other poly(C) RNA-binding proteins in nature, which utilize a loop structure as a binding surface, the CP1 peptide likely supports the interaction with polyRNAs *via* its β-sheet structure. Furthermore, the fact that polyQ/polyN peptides do not exhibit strong affinity toward polyRNA (Figure S14) clearly indicates that a tandem Gln/Asn sequence alone is not enough to support affinity toward polyRNA. Although further characterization is necessary to uncover the precise binding mechanism, our findings showcase the effectiveness of our methodology in examining variations in peptide sequences that have not been investigated in natural settings.

## Conclusions

In conclusion, our cell-free codon-restricted mRNA display approach identified a catalog of possible *de novo* ssRNA-binding peptides from a synthetic peptide library containing limited amino acid alphabets. Newly identified *de novo* ssRNA-binding peptides exhibit significant promise as versatile RNA-binding tags with a wide array of potential applications ranging from the modulation of translation and stability in targeted mRNA molecules to the labeling of specific RNA molecules within cells. Our method also offers an opportunity to design unstructured-RNA targeted drugs such as developing pharmaceutical candidates for neurodegenerative disorders. The future incorporation of genetic code expansion and codon reassignment technology^54^ will lead to the expansion of our method to further explore unnatural peptide sequence space with desired amino acid combinations, including noncanonical amino acids.

## Data Availability

All data are provided in full in the Results Section and the Supporting Data accompanying this paper.

## Supporting Information Available

Complete list of DNA and RNA oligos used in this work (Table S1); the top peptide sequences of the most enriched 30 clusters in the VMM/VRR codon library (Table S2); the top peptide sequences of the most 30 enriched clusters in the HHY codon library (Table S3); schematic overview of the codon-restricted mRNA display (Figure S1); codon translation table (Figure S2); synthesis of DNA library and addition of puromycin-DNA-tag to mRNA (Figure S3); synthesis of mRNA-peptide conjugates by *in vitro* translation (Figure S4); cDNA synthesis using the selected mRNA-peptide conjugates (Figure S5); principal component analysis of amino acid frequencies (Figure S6); thermodynamic plots of the identified RNA-binding peptides with polyRNA/DNA (Figure S7); thermodynamic plots of the identified RNA-binding peptides with hybridized RNAs (Figure S8); thermodynamic plots of AP2 peptide and its mutated variants with polyRNA (Figure S9); thermodynamic plots of CP1 peptide and its mutated variants with poly(C) RNA (Figure S10); lowest-energy structures for hnRNPK-associated RNAs (Figure S11); thermodynamic plots of CP1 peptide with hnRNPK-associated RNAs (Figure S12); proposed hydrogen bonding between Gln/Asn and adenine/cytosine (Figure S13); and thermodynamic plots of polyQ/N peptide with polyRNAs (Figure S14) (PDF)
