# Supplementary material for: De Novo Single-Stranded RNA-Binding Peptides Discovered by Codon-Restricted mRNA Display
Source: Biomacromolecules. 2023 Dec 5;25(1):355–65. doi: 10.1021/acs.biomac.3c01024 (PMC10777347; doi:10.1021/acs.biomac.3c01024)
Supplement: Supplementary file 1 — bm3c01024_si_001.pdf [file bm3c01024_si_001.pdf]

Supporting Information

De novo single-stranded RNA-binding peptides discovered by codon-restricted mRNA display

Shota Nishikawa[1,2] Hidenori Watanabe[1], Naohiro Terasaka[1], Takayuki Katoh[3], and Kosuke Fujishima[1,4*]

[1] Earth-Life Science Institute, Tokyo Institute of Technology, Ookayama, Meguro-ku, Tokyo 152-8550, Japan

[2] School of Life Science and Technology, Tokyo Institute of Technology, Meguro-ku, Tokyo 152-8550, Japan

[3] Department of Chemistry, Graduate School of Science, The University of Tokyo, Bunkyo-ku, Tokyo 113-0033, Japan

[4] Graduate School of Media and Governance, Keio University, Fujisawa, 252-0882, Japan

[*] To whom correspondence should be addressed. E-mails: fuji@elsi.jp

Supporting methods

In vitro transcription. RNA libraries were produced by *in vitro* transcription from a DNA library. ScriptMax® Thermo T7 Transcription Kit (TOYOBO; Osaka, Japan) was used for *in vitro* transcription. The transcription was carried out with 200 ng (4 pmol, 2.4×10^{12} molecules) of the linear DNA molecules according to the manufacturer's protocol. The reaction mixture was incubated at 40℃ for 2 h. The RNA transcripts were then purified using NucleoSpin® Gel and PCR Clean-up Kit (Macherey-Nagel; Düren, Nordrhein-Westfalen, Germany).

Puromycin-DNA-tag ligation. The mRNA libraries attached with a puromycin-DNA-tag were generated by the Y-ligation method[1]. Puromycin-DNA-tag labeled with fluorescein isothiocyanate (FITC) was synthesized by Japan Bio Services Co., LTD (Asaka, Saitama, Japan). Initially, the mRNA library and the puromycin-DNA-tag were partially annealed. A reaction mixture containing the transcribed mRNA (20 µg), puromycin-DNA tag (8 µM), ATP (1 mM), and T4 ligation buffer (New England Biolabs, Inc.; Ipswich, MA, USA) was then incubated at 90℃ for 30 s and cooled to room temperature. Subsequently, 3 U of T4 PNK Kinase (New England Biolabs, Inc.; Ipswich, MA, USA) and 20 U of T4 RNA ligase (New England Biolabs, Inc.; Ipswich, MA, USA) were added to the mixture and incubated at 25℃ for 30 min to ligate the 5′ end of puromycin-DNA-tag to the 3′ end of the mRNA library. Next, mRNA-tag ligation products were isolated by urea polyacrylamide gel electrophoresis. For the electrophoresis, 40 mL of a separating gel consisting of urea (8 M), polyacrylamide (6%, w/v), and 1x Tris-Borate-EDTA (TBE) was run at 50 mA for 1 h. The ligation products in the gel were detected by the fluorescence specific for FITC with a blue LED transilluminator (FUJIFILM Wako Pure Chemicals; Osaka, Japan) and excised with a clean scalpel. Finally, the mRNA tag was recovered by gel-purification.

Synthesis of mRNA-peptide conjugate by in vitro translation. mRNA-peptide conjugates were synthesized from the mRNA-tag *in vitro*. PUREfrex 1.0 (GeneFrontier Corp.; Kashiwa, Chiba, Japan) was used for the *in vitro* translation. Initially, a reaction mixture (100 µL) containing 800 ng of the mRNA tag was prepared according to the manufacturer's protocols. The reaction mixture was incubated at 37℃ for 30 min. Then, the salt mixture was added to the reaction to bring its final concentration to $MgCl_2$ (32.5 mM) and KCl (375 mM). Following that, the reaction mixture was

incubated again at 37℃ for 1 h. An equal amount of 2x Laemmli sample buffer (Bio-Rad; Hercules, CA, USA) was then added to the reaction and centrifuged at 10,000 × g for 3 min to remove the precipitants. The supernatant was collected and loaded on a SDS-Urea-PAGE gel (3.5% w/v SDS-PAGE stacking gel, 10% w/v SDS-6 M Urea-PAGE resolving gel). The electrophoresis was conducted at 30 mA for the stacking gel and at 50 mA for the resolving gel. Note that a 4–12% w/v gradient SDS-PAGE gel (TEFCO; Hachioji, Tokyo, Japan) were used from round 3 to round 6 of the VMM/VRR codon libraries instead of the SDS-Urea-PAGE gel, since we could separate the mRNA-tag and mRNA-peptide conjugates with higher resolution. For a 4–12% w/v gradient SDS-PAGE gel, electrophoresis was conducted at 60 mA for 90 min. Finally, the mRNA-peptide conjugates were isolated by excision as previously described[2], and purified by gel-purification.

Gel-purification. A gel slice containing the desired band was purified from a gel through electroelution followed by ethanol precipitation. For electroelution, Model 422 Electro-Eluter (Bio-Rad; Hercules, CA, USA) apparatus was used along with a glass tube, a frit, and a membrane cap (MWCO 12 kDa) from Bio-Rad. After this apparatus was properly assembled, the kneaded gel slice was loaded into the glass tube filled with 1x TBE buffer. The sample was eluted at 10 mA/glass tube for 45 min. At the end of elution, the polarity of the initial current was reversed for 30 s to dissociate the purified products from the dialysis membrane. Then, the eluate was carefully collected into the microcentrifuge tube. As for ethanol precipitation, sodium acetate (3 M) equivalent to 10% v/v of the obtained volume was added to the eluate and mixed well. A volume of pre-chilled ethanol (99.5%, v/v) (FUJIFILM Wako Pure Chemical; Osaka, Japan) equal to three times the total sample was further added to the mixture. The sample mixture was then centrifuged at 4℃, 15,000 × g for 1 h. After removing the supernatant, 1 mL of the chilled 70% v/v ethanol was added to the microcentrifuge tube. The dislodged pellet in ethanol (70%, v/v) was centrifuged again at 4℃, 15, 000 × g for 15 min. Subsequently, the supernatant was removed, and the pellet was air-dried at room temperature to evaporate the residual ethanol. Finally, the pellet was resuspended in nuclease-free water and the mRNA concentration was measured with NanoDropTM2000c spectrophotometer (Thermo Fisher Scientific; Waltham, MA, USA).

).

Negative selection of mRNA-tag by the affinity with poly RNA. Negative selection was performed before *in vitro* translation to eliminate any mRNA products that accidentally obtained complementary bases *via* PCR or transcriptional error. First, 20 µL of 25-µm strep-tactin-immobilized magnetic beads (MagStrep "type3" XT Beads; IBA Lifesciences; Göttingen, Germany) was captured by a magnetic stand (DynaMag-2 Magnet; Thermo Fisher Scientific; Waltham, MA, USA) and washed twice with RNA-binding buffer (Tris-HCl (10 mM; pH 7.4), EDTA (0.5 mM), NaCl (500 mM), Tween 20 (0.05%, v/v)). After that, the poly RNA was immobilized on the magnetic beads. Biotinylated poly RNA (2.5 µM) in 200 µL of the RNA-binding buffer was added to the magnetic beads and mixed at 4℃ for 30 min with a rotator (80 rpm). After discarding the supernatant, the magnetic beads were washed three times with the RNA-binding buffer. Subsequently, 25 µL of the mRNA-tag (100 ng/µL) in the RNA-binding buffer (Tris-HCl (10 mM; pH 7.4), EDTA (0.5 mM), NaCl (500 mM), Tween 20 (0.05%, v/v)) was added to the magnetic beads and mixed for 30 min at room temperature with a rotator (80 rpm). Finally, the supernatant was collected for the following *in vitro* translation.

Peptide selection based on the affinity with poly RNA. The mRNA-peptide conjugates with affinity to poly RNA were selected over the affinity of its mRNA-tag portion. First, two sets of strep-tactin-immobilized magnetic beads (20 µL) were washed twice with the RNA-binding buffer by magnetic stand. Next, the target poly RNA was immobilized to one of those magnetic beads as described in the previous section. Subsequently, 100 ng of mRNA-peptide conjugates (1.7 pmol, 1.0×10^{12} molecules) was prepared in the RNA-binding buffer (200 µL; Tris-HCl (10 mM; pH 7.4), EDTA (0.5 mM), NaCl (500 mM), Tween 20 (0.05%, v/v)), and the solution was first added to the intact magnetic beads and mixed for 10 min at room temperature with a rotator (80 rpm) to remove the conjugates with affinity to the surface of the beads. To avoid the effect of metal cations on the peptide-RNA interaction, EDTA was added to the RNA-binding buffer. The supernatant, containing mostly the nonspecific binding-free mRNA-peptide conjugates, was then recovered and used for the affinity selection with the target RNA. The entire supernatant was added to the target RNA-immobilized magnetic beads and mixed for 30 min at room temperature with a rotator (80 rpm). After the removal of the supernatant, the magnetic beads were washed three times with 1x Buffer W (IBA Lifesciences; Göttingen, Germany). The 25 µL elution buffer (Tris-HCl (100 mM; pH 8.0), NaCl (150 mM), EDTA (1 mM), Biotin (50 mM)) was then added and vigorously mixed.

Finally, the mixture was incubated for 10 min at room temperature. The eluate was collected and used for reverse transcription polymerase chain reaction (RT-PCR).

Reverse transcription PCR (RT-PCR). The eluate mRNA-peptide conjugates (10 µL) were used as a substrate for RT reaction using ReverTra Ace-α- (TOYOBO; Osaka, Japan) with RT-PCR-F and RT-PCR-R primers (each 0.2 µM) by following the manufacturer's protocol. RT reaction (20 µL) was started by adding the reverse transcriptase (reverse transcription reaction conditions: 50℃, 20 min, 99℃, 5 min, and 4℃, 5 min). The reaction was then mixed with 20 µL of Q5 High-Fidelity 2x Master Mix (New England BioLabs; Ipswich, MA, USA) and aliquoted into a total of six tubes to recover samples at different PCR cycles (0, 10, 15, 20, 25, and 30 cycles) to check the amplification efficiency. PCR condition was set as follows: initial denaturation at 98℃ for 30 s, followed by 30 cycles of 98℃ for 10 s, 65℃ for 10 s, and 72℃ for 10 s, then final extension at 72℃ for 30 s. To avoid over-amplification of DNA, these reaction samples were run on a 3% w/v TAE agarose gel stained by SYBR Gold, and the optimal amplification cycle was determined based on the band intensity and the absence of non-specific amplified products. Finally, RT-PCR reaction (40 µL) was once again conducted with optimal PCR cycles, and the product was purified using NucleoSpin™ Gel and PCR Clean-up Kit (Macherey-Nagel) for the next round of selection and sequencing.

Sequence analysis. Sequence files were obtained from the MiSeq Illumina platform as FASTQ files. A self-made pattern search program written in Perl script was used to extract the coding region, which is the region between the upstream of the start codon and the fixed sequence (8 nucleotide sequence) of the random region. Then, by using the count option of the software called FASTAptamer[3], the number of identical sequences was counted and ranked for each round. Since the number of reads acquired in each round was different, we followed the progression of enrichment based on the normalized reads per million (RPM) value. For round 7, we performed clustering analysis on peptide sequences with RPM values of 10 or higher to create clusters based on their sequence similarity. In this case, we used the cluster option of FASTAptamer to define a cluster as a group of peptide sequences with a distance of 7 or less between peptide sequences. The sequence logo of the selected RNA-binding peptides was created using WebLogo 3 server[4],

and log2-fold changes of amino acid frequencies after selection was calculated from the average amino acid frequencies in round 7 and 0.

Principal component analysis. The amino acid frequencies within each peptide were calculated by a self-made program written in Perl script. Principal component analysis was performed against the calculated amino acid frequencies using R ver 4.0.2 (https://www.r-project.org/), and the loading plots were drawn using the first and second principal component axes. The peptide sequence dataset was prepared from randomly collected 100 peptide sequences from the peptide sequences in round 0 and the top peptide sequences of the top 30 enriched clusters in round 7.

Calculation of hydrophobicity and net charge of peptides. The hydrophobicity and net charge index of peptides were calculated using "Peptides" ver 2.4.3 package 50 of R ver 4.0.2[5]. The GRAVY score (Grand average of hydropathy), which is a hydrophobicity index of peptides, was calculated based on Kyte and Doolittle’s amino acid hydrophobicity index[6]. The net charge index at pH 7.4 was calculated using the Henderson-Hasselbalch equation using Lehninger’s pKa scale[7].

Enrichment analysis of combinatorial amino acid pairs. The frequency of combinatorial dipeptide motifs of peptide libraries from round 0 and round 7 was calculated, and the enrichment score was derived from the following equation. Hierarchical clustering was then used to visualize the enrichment score of dipeptide motifs by heat mapping.

$$Enrichment\ score\ (\%) = \frac{Average\ frequency\ of\ dipeptide\ motif\ in\ round\ 7 - Average\ frequency\ of\ dipeptide\ motif\ in\ round\ 0}{Average\ frequency\ of\ dipeptide\ motif\ in\ round\ 0} \times 100$$

Microscale thermophoresis Chemically synthesized fluorescein (FAM)-labeled CP1 peptide was obtained from genscript (Piscataway, NJ, USA). The oncogenic RNA motifs were synthesized by *in vitro* transcription described in the previous section. The RNA transcripts were then purified using Monarch RNA Cleanup Kit (New England Biolabs, Inc.; Ipswich, MA, USA). Microscale thermophoresis experiment was conducted with a set of biologically derived RNAs in three independent experimental trials on a Monolith NT.115 (NanoTemper Technologies; Munich, Bayern, Germany) unless specified. Both the FAM-labeled CP1 peptide and RNA were prepared in Human Plasma-Like Medium (Gibco; Billings, MT, USA) supplemented with 0.1 mg/mL bovine serum albumin (Sigma; St. Louis, MO, USA). A two-fold dilution series of the unlabeled

RNAs were prepared in 50 nM FAM-labeled CP1 peptide, with the final concentrations of RNAs ranging from 2 μM to 0.06 nM. Samples were incubated for 30 min at room temperature. Following incubation, the samples were filled into standard treated capillaries (NanoTemper Technologies; Munich, Bayern, Germany) and subsequently subjected to microscale thermophoresis. General settings were applied for all microscale thermophoresis experiments as follows: manual temperature control: 25℃, LED laser: blue, fluorescence measurement before microscale thermophoresis: 5 s, microscale thermophoresis (IR laser) on: 45 s, fluorescence after microscale thermophoresis: 15 s, delay: 25 s. LED and microscale thermophoresis power settings were chosen individually for each sample by adjusting the LED power to yield fluorescence signals of at least 200 units and to achieve an appropriate thermophoretic response. The values obtained were normalized and plotted against the peptide concentration. The dissociation constant was then determined using a single-site model to fit the curve. Microscale thermophoresis was also employed to determine the dissociation constants between polyQ/N peptide and (Cy5)-labeled oligonucleotides. The polyQ/N peptide (6-mer) was chemically synthesized and purified with high purity (>90%) by BEX (Itabashi, Tokyo, Japan). The oligonucleotides were prepared in a buffer containing Tris-HCl (20 mM; pH 8.0) and Tween 20 (0.1%, v/v), and the peptide were prepared in RNase-free water. A two-fold dilution series of the peptides were prepared in 20 nM oligonucleotide, with the final concentrations of peptides ranging from 500 μM to 15 nM. The following measurement and analysis were conducted as described above.

Supporting Tables

Table S1. Complete list of DNA and RNA oligos used in this work.

Name	Sequence (5′ to 3′)
PolyAGCU-binding-F	GGCGTAATACGACTCACTATAGGGTTAACTTTAACAAGGAGAAAAACATG
(HHY)n-library-R	TTTTTCACCTGATCCGCTGCTGGGTGA**RDDRDDRDDRDDRDDRDDRDDRDDRDDRDD**<br>**RDDRDDRDDRDDRDDRDDRDDRDDRDDRDDRDDRDDRDDRDDRDDRDDR**<br>**DDRDDRDD**CATGTTTTTCTCCTTGTTAAAG<br><br>*D = A/G/T, R = A/G
(VMM/VRR)n-library-R	TGGTTCACCTGATCCGCTGCTGGGTCC**KKBYYBKKBKKBYYBKKBYYBKKBYYBYYBKKB**<br>**YYBKKBKKBYYBKKBYYBKKBYYBYYBKKBYYBKKBKKBYYBKKBYYBKKBYYBYYBKK**<br>**BYYBKKB**CATGTTTTTCTCCTTGTTAAAG<br><br>*B = C/G/T, K = G/T, Y = C/T
RT-PCR-F (PolyAGCU-binding-F)	GGCGTAATACGACTCACTATAGGGTTAACTTTAACAAGGAGAAAAACATG
RT-PCR-R	TTTTTCACCTGATCCGCTGC
Puromycin-DNA-tag	pCCCTTCACCTGATCCGCTGAAAAAAAAAAAAAAAAAA (Spacer 18) (Spacer 18) (F-dT) (Spacer 18) CC (Puro)<br><br>(Spacer 18): Hexaethylene glycol,<br>(F-dT) = Fluorescein-dT,<br>(Puro) = Puromycin,
5′ biotin-Poly(C) RNA	(Biotin)-CCCCCCCCCCCC
5′ biotin-Poly(A) RNA	(Biotin)-AAAAAAAAAAAA
5′ Cy5-Poly(A) DNA/RNA	(Cy5)-AAAAAAAAAAAA
5′ Cy5-Poly(C) DNA/RNA	(Cy5)-CCCCCCCCCCCC

Table S2. The top peptide sequences of the most enriched 30 clusters in VMM/VRR codon library. The top peptide sequences were listed as a representative of clusters.

Cluster No.	Representative sequence	Unique sequences	RPM
1	MEEAKRTKTEEPKTQRHEEKQEQQKQPRHQATRP	19	15079.0
2	MPQPKQPQNQPERDQKNQNQAPQKQGTGARQKRP	10	12055.7
3	MDKEGGTQPRHTRAEKKENQTQQNARDRNQHQRP	4	6304.6
4	MPRNQQQKQKPARDQKTEHQNTRERGQRPETHRP	6	6644.2
5	MHKTEENQPKQHQHQEPKEQAHKTRGTRPRAQGN	4	5713.6
6	MNEDKQHRHKNTETKQPRTQTTRPQGPENETAKP	2	5182.2
7	MPEPKKAKQEKPEPEQTKPENKGAGQPRHQTARP	4	4862.4
8	MHQQKQTETETAKPRQHEPKTPQPERQRDRAKEH	3	4405.9
9	MPKNKQKRNQTKQDQKHEEQHQQTQKPQNQAHQN	4	4412.6
10	MARPRQHRPEPHRPEETGDETKQHQRDQNQQPGA	2	4156.8
11	MQKTQEPKDQEAQHQRQQKEQHRDQRPGTREHQP	2	3671.6
12	MTKHQQHQEQATRPQRNRNQKARPQGKRTEDKRP	6	3389.4
13	MTQEEKPQTRQNRHQKTQTQPPGEREHGAQHTEP	3	3155.6
14	MQEEEKTKKEEHEPKRPQQEPKQKRQHQPRDAGT	3	3144.6
15	MTREKKQKEQPAQQQKHQNQQPKTEGHQERHHKT	1	2968.2
16	MTQPERKRQETKEHKQTKHKETEHQGDRTKHTRD	5	3076.2
17	MAQEGRQEHQKTKAQQEKHRKDQQEETKHQAARP	1	2738.8
18	MTQNQRTKTQAPGDRENRPQKHENKQTETQTPQP	1	2652.8
19	MTKTKRTRNQDNKDQKPKDEAPQPKEHGPEAQGP	2	2661.7
20	MNEQQKQQTRTPETGQTKHENNQPKRQRTQNQRP	4	2641.8
21	MHKKEQPRDRTPENQQNQKQHTRNERARTKPPRP	2	2580.1
22	MHEDKKDEPQEQKHEKPRTQKNQTERKEDRAQEP	2	2549.2
23	MTRHQQHKTQTDEHEREEDETKRDRRPENRPART	2	2333.1
24	MTQPQKNQHRPNGTKRQEHGPNEHQQKRARAPQP	2	2293.4
25	MTQPKRTRPQNHQQKQNKPQNNRTRRKEPKEQEA	3	2255.9
26	MTEEERTQTKTERHQRPGDRTQQTKEHQHRDNKT	2	2220.6
27	MGKARRTKTEQQQAKRKENQHHQTEQQQHQHHRT	1	2123.6
28	MHEDQKQQKQHTREEQTKPEPKQTKQHRTRAQQD	4	2158.9
29	MEGAGRKGPQKTKTQETREKHAKHERTEHRPART	2	1991.3
30	MNQHQQTKTEDQRPGRTRAEQAQTREDRERHARP	1	1947.2

Table S3. The top peptide sequences of the most enriched 30 clusters in HHY codon library.

The top peptide sequences were listed as a representative of clusters.

Cluster No.	Representative sequence	Unique sequences	RPM
1	MTHNHNYHHHITNNNINHTNPNNYTITNSPNNIY	362	775655.1
2	MTIFNTNHTNLYNNHTSNNNSTNSHTHHTPSSTT	37	14581.5
3	MHNNSNSNHTINNNTHHTTNNFILINYSYYHYHT	33	14426.1
4	MFNHYHTNNNTHNNHTNIHTTTHTINNPNHFYLL	21	9245.6
5	MGGPAAVLRGGAAVLVLVLLLVLLLVLALAPLAA	18	8783.1
6	MTFNYTTTHTTTTYNHYTTTNHHTNYHTSPYLYF	5	3349.5
7	MNYINHYNNHNNSNHNITNHNTLNHTPHLFFLFP	1	2597.2
8	MSYPYHTNTYNNIHNFPPFNHSNTYIYNTNHSLH	1	1131.1
9	MTSNFYHFYPHNNNHTNNTNHTILSNHNNYINHT	1	605.7
10	MHHTNSTSIYNIYNPTFYNSTNLHNTNNHYTNTN	1	549.8
11	MTNYTNTTHTNHNPPTNNNPYYTHFYTNLSPTNI	1	268.8
12	MYHINIIPINYTNNSNTNTNHYTLNNFINNTNHS	1	225.2
13	MINTFFNNSYTNTNTPYNNNTHNHTNNYTNPTHL	1	212.9
14	MHTNPTHPNNITSNSNHNNHINNNHSYYINSSYH	1	157.1
15	MTNTNTNHTNHNYNSYTTSNTNQHNYLLYILFNS	1	155.3
16	MYYIINNHPNNHYSNNTNNNHPSNNTINNLYYTT	1	141.4
17	MNNLNTIYPTTNHTYTNNHNHNTTNYTHHISHNN	1	129.2
18	MNHNIPIYNLTNNNTTTNFHNHNTNSNHNFIILH	1	120.4
19	MTFIHNHHHHHTNINHTNNSNIHNTTHSFFFPSL	1	113.5
20	MNNNTIFTHFNSHTNLNNNNNHNHNPISFFHTTH	1	106.5
21	MNYNSIIHNNNNHTTHFHTNNLNSTNNFIIHTNF	1	104.7
22	MLNNHINIYHHTTYIPNYNSNNNSHHTSIYFPST	1	97.7
23	MIHNSIHYTNTNPYNHTNSNITNYSNHTLFPTLH	1	90.8
24	MLNNHHTNHITTTLNHTNNNSNTIINSTYTIHNT	1	52.4
25	MTHNPIINSNYNYNYPFYNNNLTLNNHTNHNHTT	1	50.6
26	MHNSNNHTSYNHTHNILFNNNNSNNNNHTNLLHN	1	48.9
27	MNYFNYTNINNLNNHNNLTNHHPPNPPFTLNSFH	1	48.9
28	MTNTNTNHTNHNYNSYTTSNTNNTIIFFTFSSNS	1	41.9
29	MIFHYHTHNNYNTTSNLNYNHTNHIIHTSTLFHN	1	38.4
30	MTNYYINTHINNHTNYSNTTNPTSPLFHNIFHTS	1	34.9

Supporting Figures

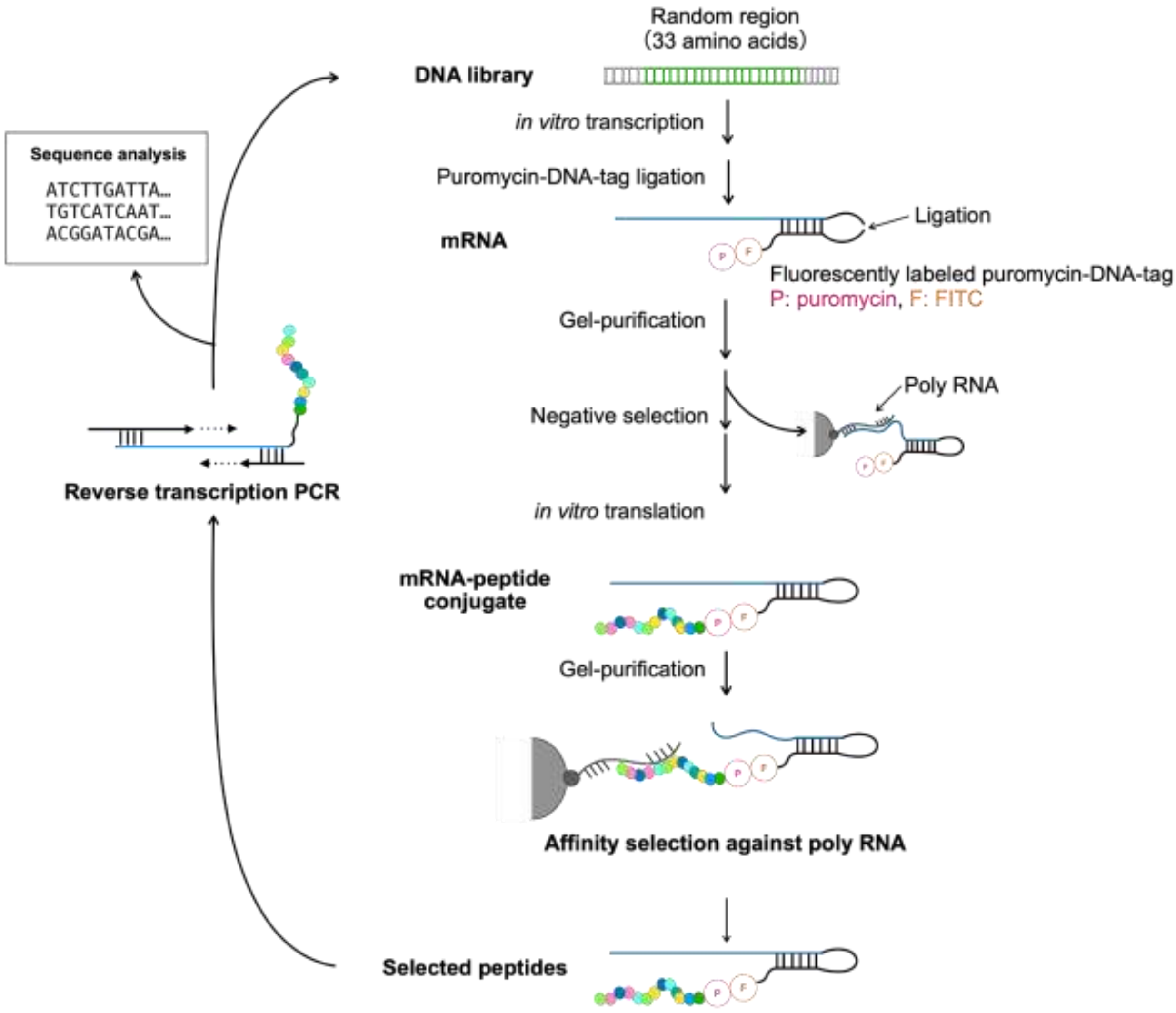

Figure S1. Schematic overview of codon-restricted mRNA display. This method starts with a designed codon-restricted DNA library (VMM/VRR or HHY codon library) encoding 33-amino acid random peptides. The DNA library is *in vitro* transcribed to mRNA library and undergoes ligation with the puromycin-DNA-tag. The tagged products are gel-purified and subjected to negative selection. The remaining tagged products are *in vitro* translated using a cell-free system and the resulting mRNA-peptide conjugates with a variety of 10^{12} sequences are gel-purified and subjected to affinity selection against poly RNA. The selected peptide-mRNA conjugates are reverse-transcribed to the DNA library and used in the next round of selection. Also, DNA libraries from each round (round 0 to round 7) are subjected to Illumina MiSeq sequencing.

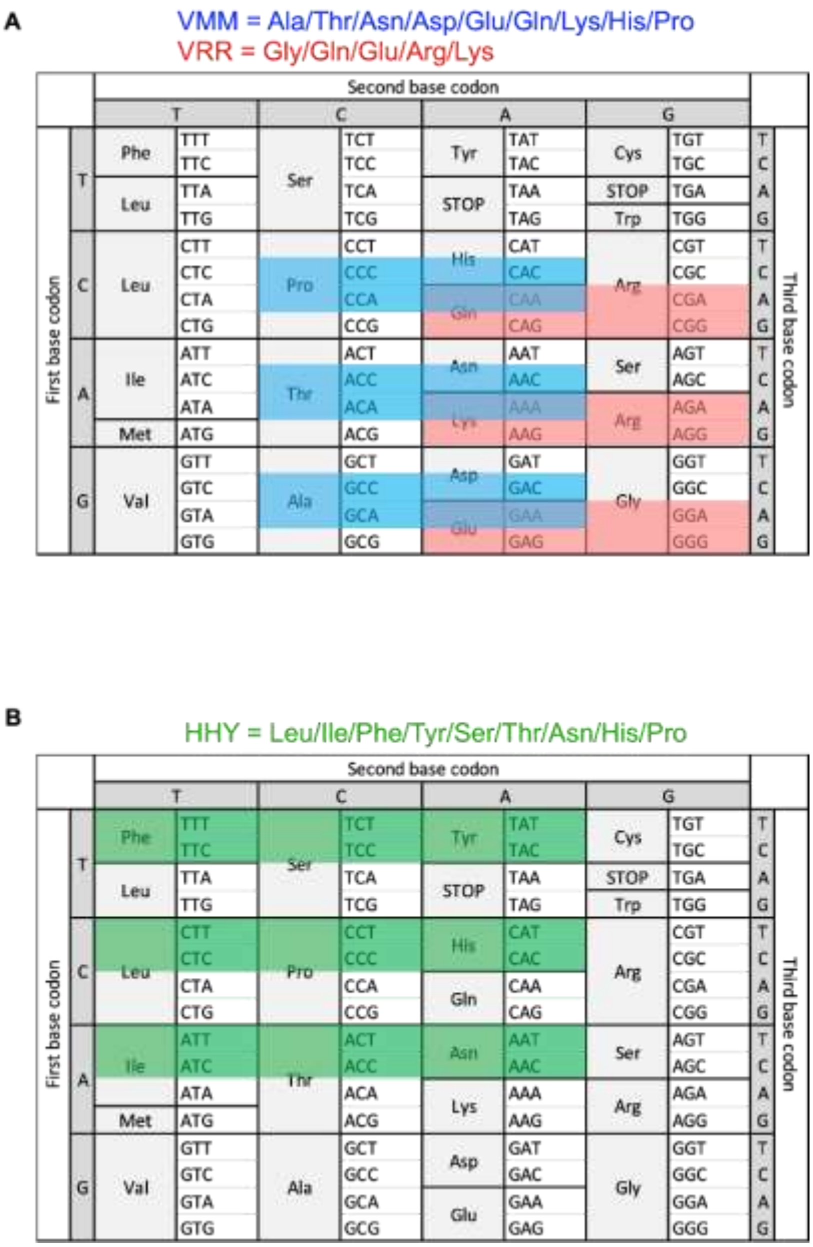

Figure S2. **Codon translation table.** The codons used by each library are shown in the codon table. **(A)** Codons corresponding to "VMM" and "VRR" in the VMM/VRR codon library are highlighted in blue and red, respectively, while **(B)** codons corresponding to "HHY" in the HHY codon library are highlighted in green.

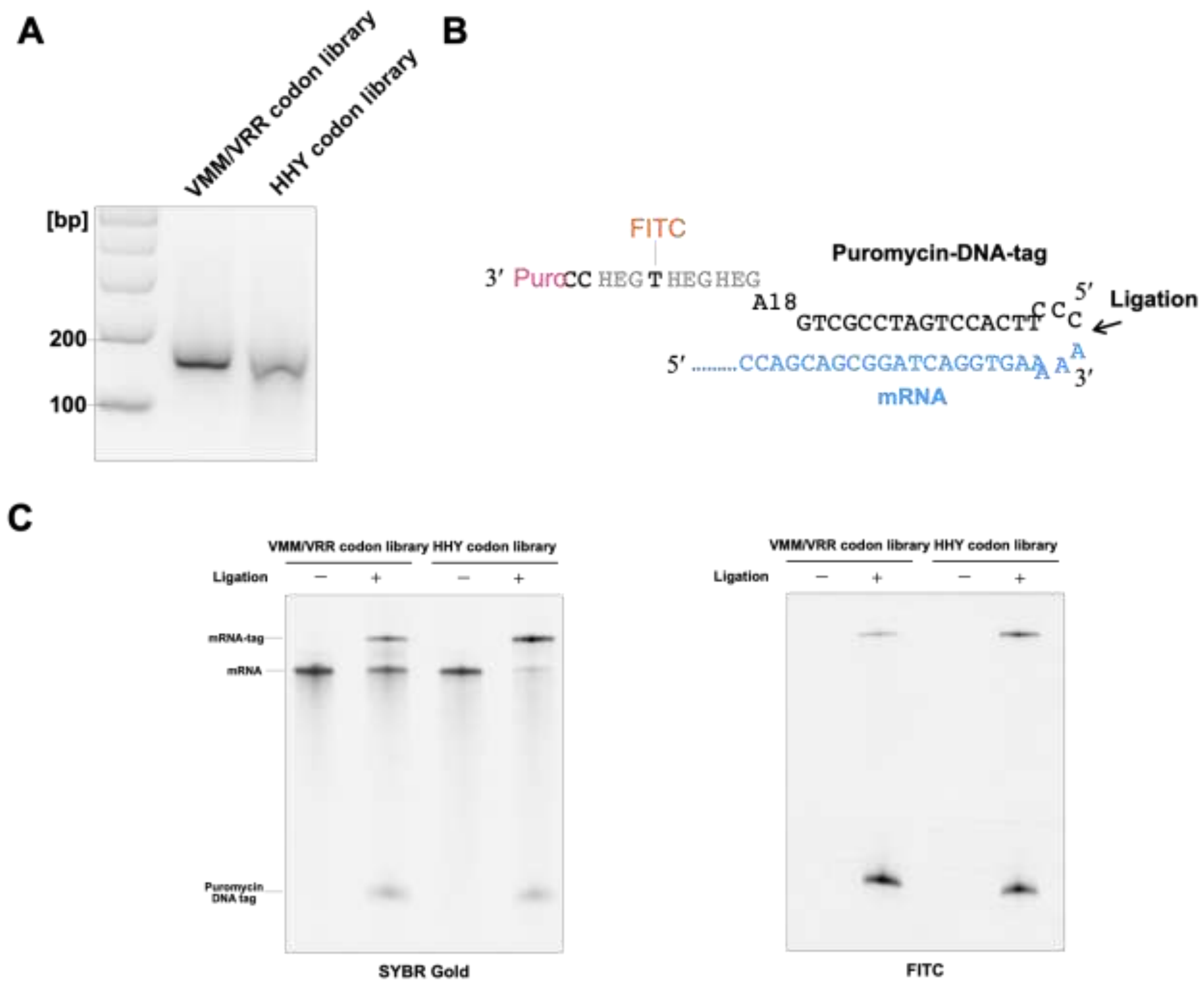

Figure S3. **Synthesis of DNA library and addition of puromycin-DNA-tag to mRNA. (A)** Gel images were taken after synthesis of the DNA libraries and electrophoresis in a 3% w/v TAE agarose gel. **(B)** Schematic overview of Y-ligation reaction. **(C)** Gel photograph of mRNA and Y-ligation reaction mixture after electrophoresis on a 8 M urea 6% w/v TBE gel (left: SYBR Gold staining, right: FITC fluorescence detection).

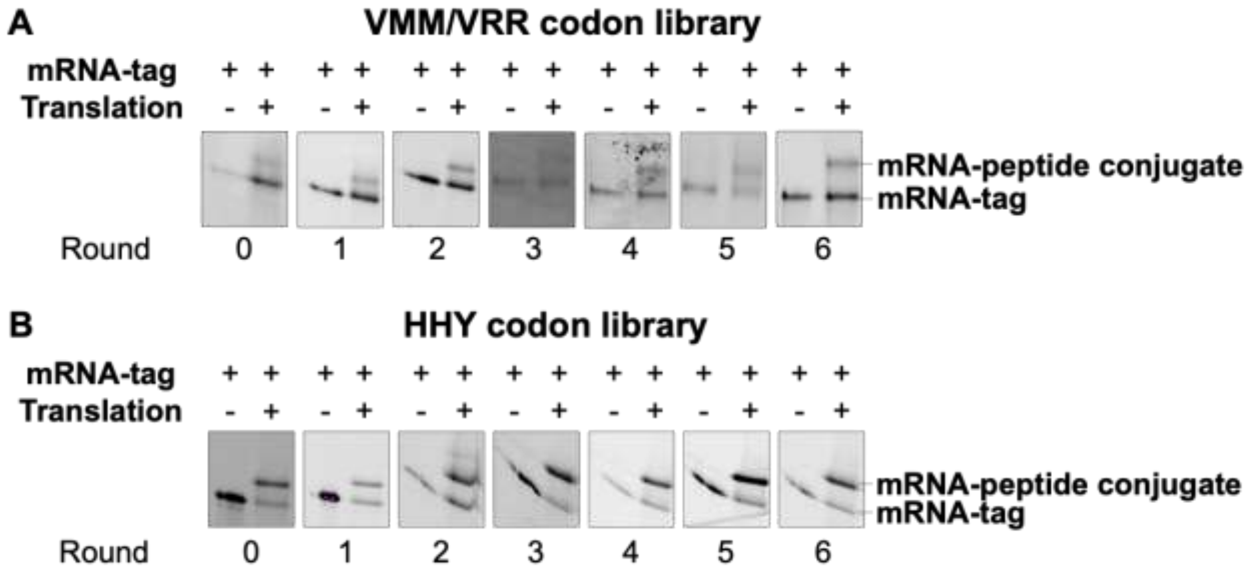

Figure S4. Synthesis of mRNA-peptide conjugates by *in vitro* translation. *In vitro* translation reactions were performed with puromycin-DNA-tagged mRNA synthesized in each round and electrophoresed on SDS-polyacrylamide gels (stacking gel: 3.5% w/v polyacrylamide gel, separation gel: 10% w/v polyacrylamide gel) (For the VMM/VRR codon library, 4–12% w/v polyacrylamide gels were used from round 3 onwards). Subsequently, the images were captured by FITC fluorescence: **(A)** VMM/VRR codon library and **(B)** HHY codon library.

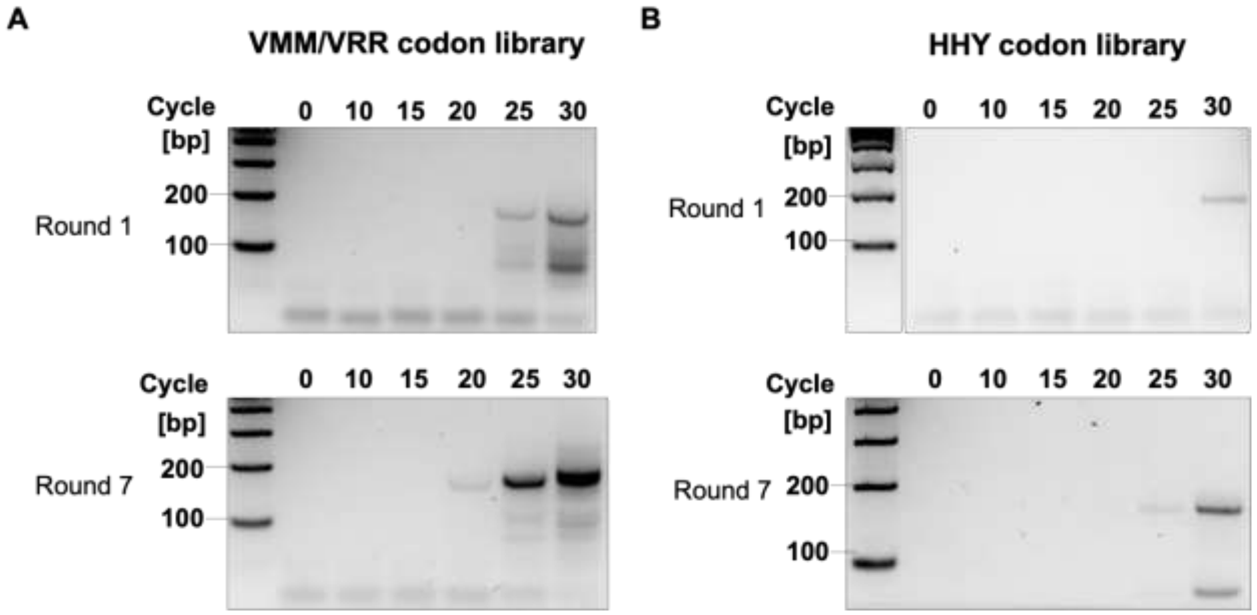

Figure S5. cDNA synthesis using the selected mRNA-peptide conjugates. cDNAs were synthesized from the mRNA-peptide conjugates selected by their targeted RNA binding capability. mRNA-peptide conjugates were reverse transcribed and amplified by polymerase chain reaction. The synthesized cDNA was electrophoresed on a 3% w/v TAE agarose gel: **(A)** VMM/VRR codon library and **(B)** HHY codon library.

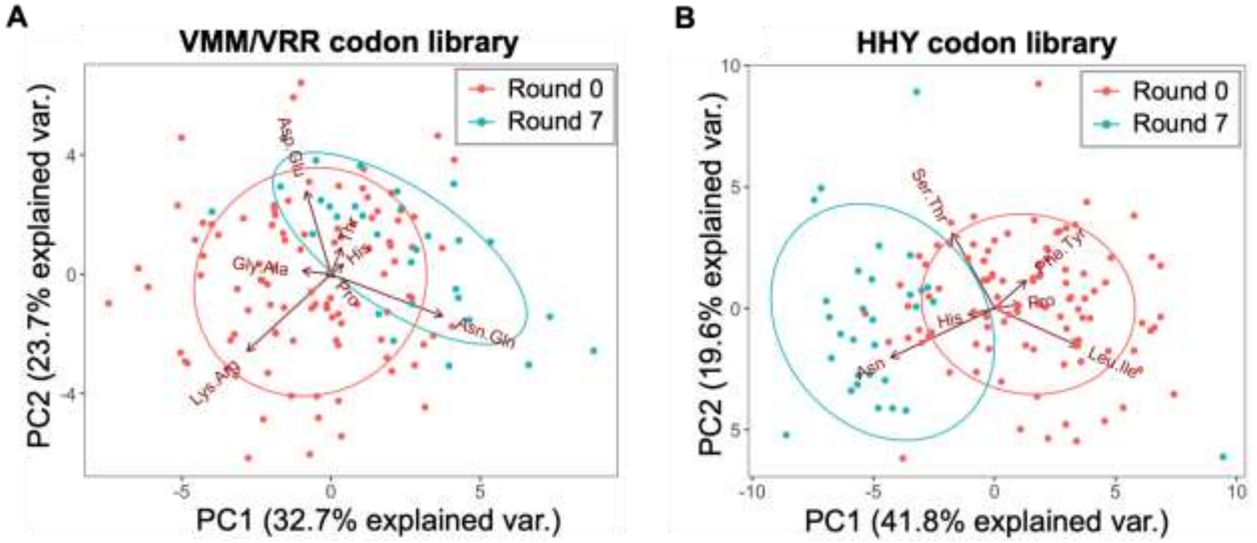

Figure S6. Principal component analysis of amino acid frequencies. For round 0, 100 peptide sequences were randomly selected, while for round 7, the representative peptide sequences of the

top 30 enriched clusters were used for analysis: **(A)** VMM/VRR codon library and **(B)** HHY codon library. Horizontal axis: first principal component, vertical axis: second principal component.

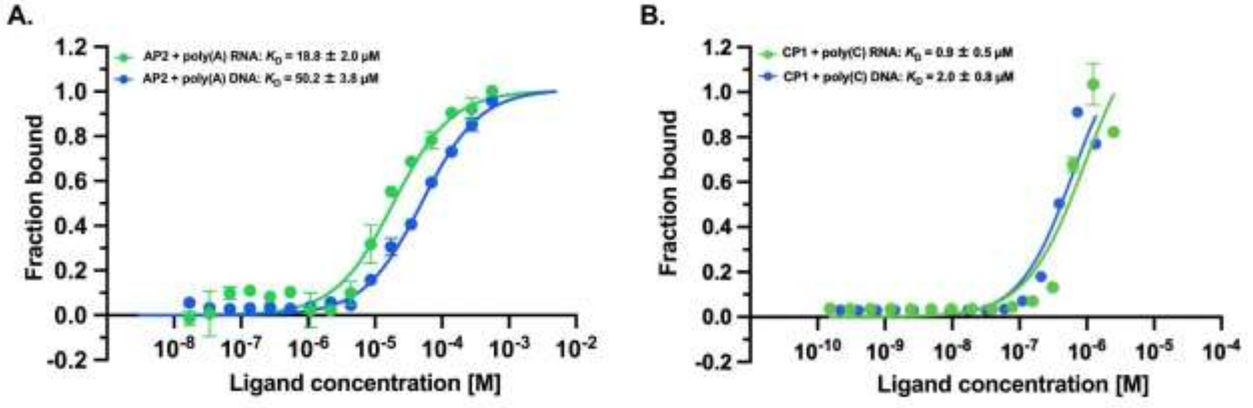

Figure S7. Thermodynamic plots of the identified RNA-binding peptides with polyRNA/DNA. The dissociation constants between polyRNA/DNA and AP2/CP1 were determined by spectral shift (SpS) experiments; **(A)** thermodynamic plots of AP2 against poly(A) RNA/DNA, **(B)** thermodynamic plots of CP1 with poly(C) RNA/DNA. The error bar represents the mean ± SE from three independent experimental replicates.

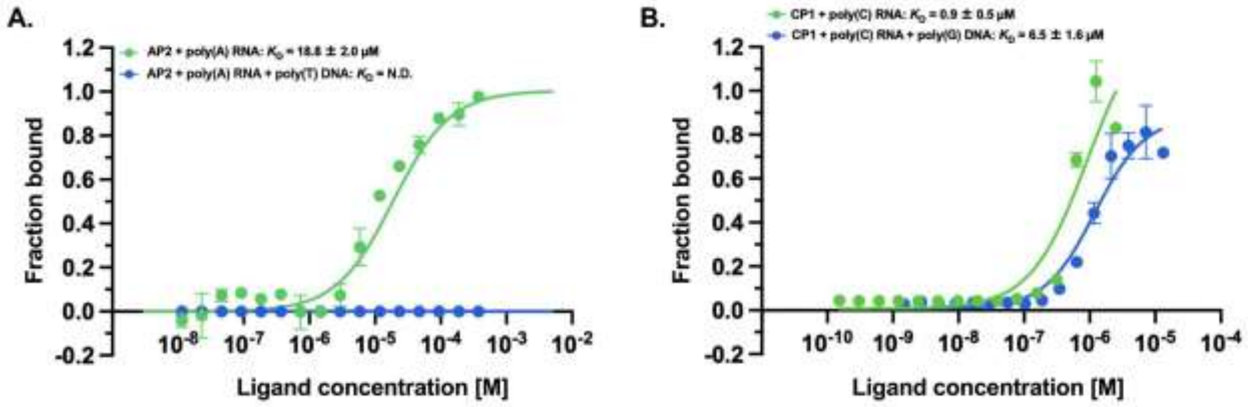

Figure S8. Thermodynamic plots of the identified RNA-binding peptides with hybridized RNAs. The dissociation constants between poly RNA and AP2/CP1 were determined in the presence of the antisense oligo DNA by spectral shift (SpS) experiments: thermodynamic plots of **(A)** AP2 with cy5-labeld poly(A) RNA in the presence of poly(T) DNA and **(B)** CP1 with cy5-labeled poly(C) RNA in the presence of poly(G) DNA. The error bar represents the mean ± SE from three independent experimental replicates.

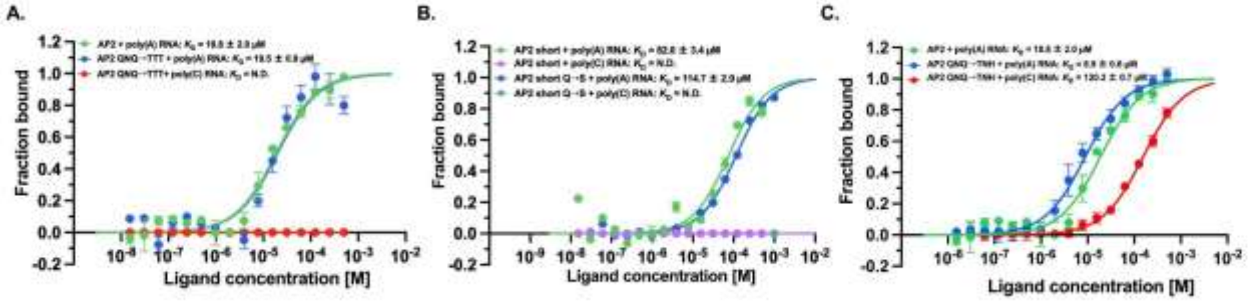

Figure S9. Thermodynamic plots of AP2 peptide and its mutated variants. The dissociation constants between AP2 variants and poly(A)/(C) RNA were determined by spectral shift (SpS) experiments. Native peptide (AP2) was compared to the mutated variants whose QNQ sequence was mutated to **(A)** TTT or **(C)** TNH, while **(B)** the truncated AP2 (position 6 to 26) was compared to the mutated variants whose Q was mutated to S. The error bar represents the mean ± SE from three independent experimental replicates.

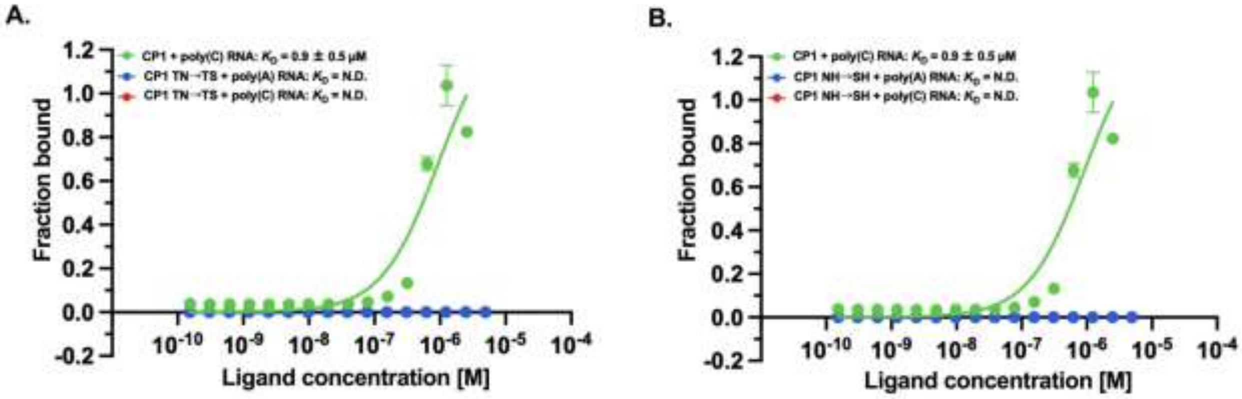

Figure S10. Thermodynamic plots of CP1 peptide and its mutated variants with poly(C) RNA. The dissociation constants between CP1 variants and poly(C) RNAs were determined by spectral shift (SpS) experiments. Native peptide (CP1) was compared to the mutated variants whose **(A)** TN sequence was mutated to TS or **(B)** NH sequence was mutated to SH. The error bar represents the mean ± SE from three independent experimental replicates.

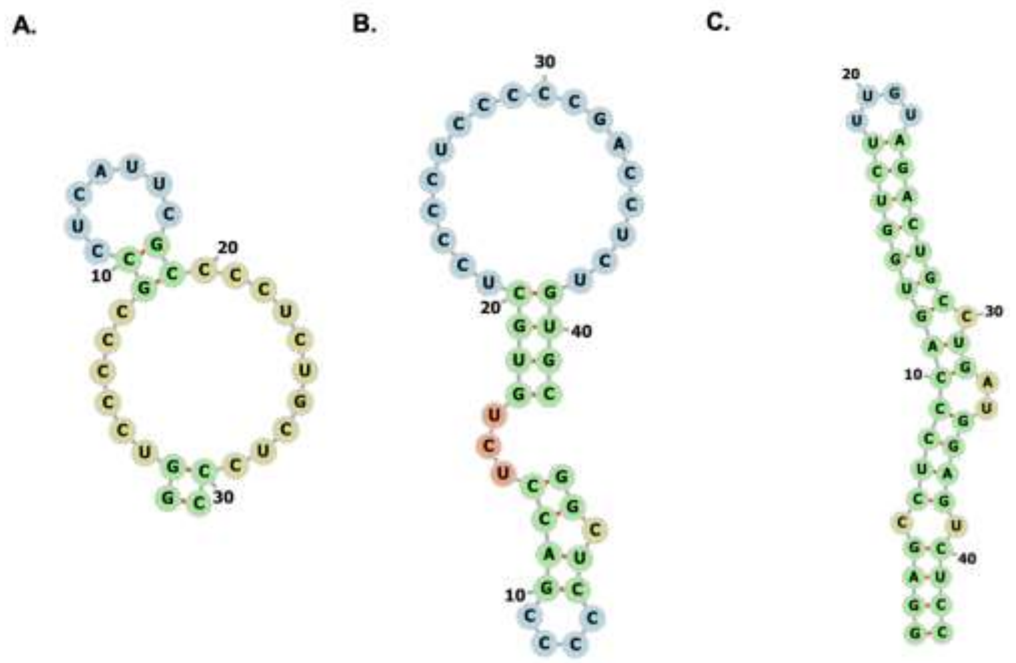

Figure S11. Lowest energy structures for hnRNPK-associated RNAs. MXfold server[8] was used to predict the lowest energy structures for hnRNPK-associated RNAs used in this study: **(A)** Cdk6 3′UTR RNA, **(B)** MYU lncRNA, and **(C)** Gas5 hp RNA.

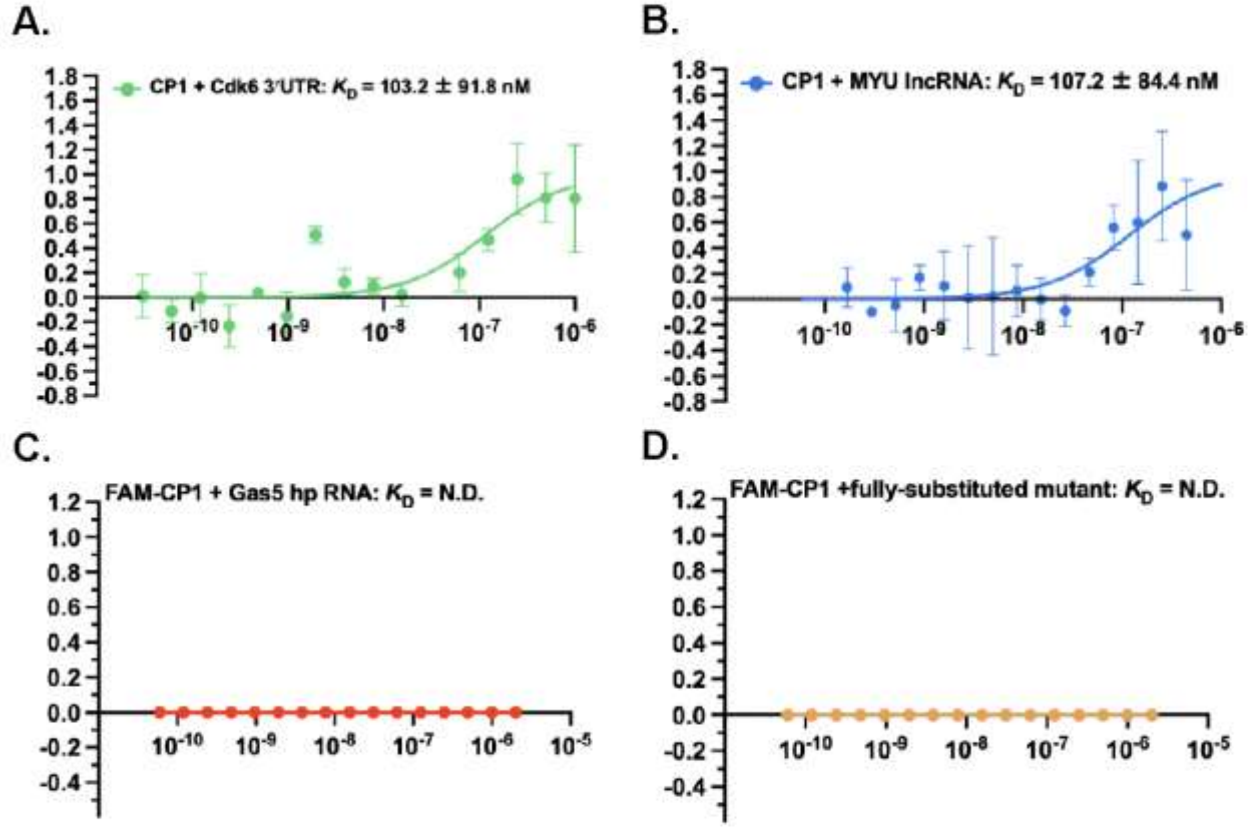

Figure S12. Thermodynamic plots of CP1 peptide with hnRNPK-associated RNAs. Microscale thermophoresis was employed to determine the dissociation constants of CP1 with a set of hnRNPK-associated oncogenic RNA motifs including hnRNPK-nonassociated RNA (Gas5 hp RNA) and fully-substituted mutant of Cdk6 3′UTR RNA. **(A)** Cdk6 3′UTR RNA, **(B)** MYU lncRNA, **(C)** Gas5 hp RNA, **(D)** fully-substituted mutant of Cdk6 3′UTR RNA. The error bar represents the mean ± SE from three independent experimental replicates.

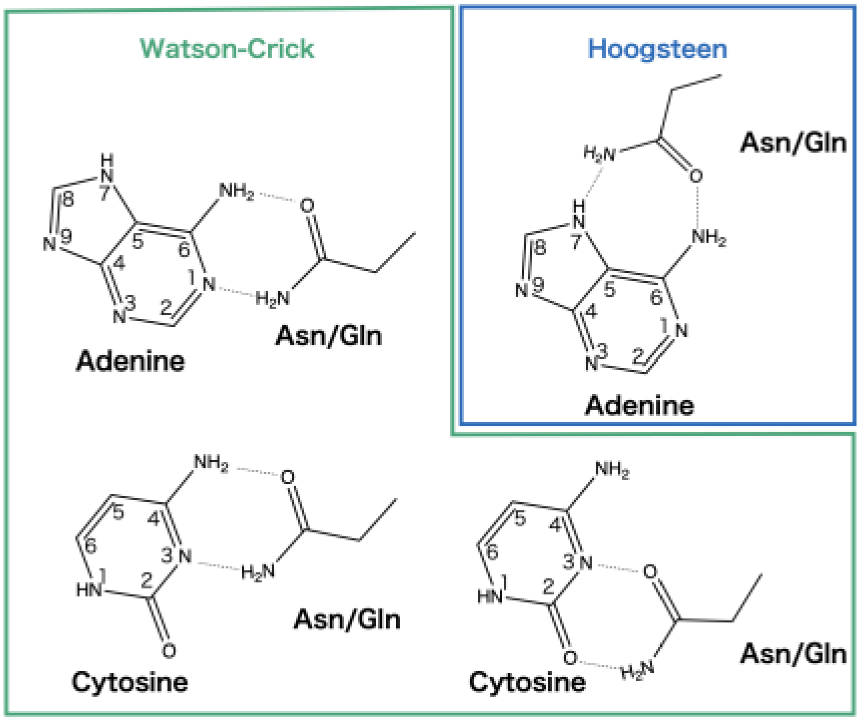

Figure S13. Proposed hydrogen bonding between Gln/Asn and adenine/cytosine. Green: Watson-Crick-type hydrogen bonding interaction between Gln/Asn and adenine/cytosine. Blue: Hoogsteen-type hydrogen bonding interaction between Gln/Asn and adenine. The common side chain structure of Gln and Asn is shown here. The dashed line indicates hydrogen bonding.

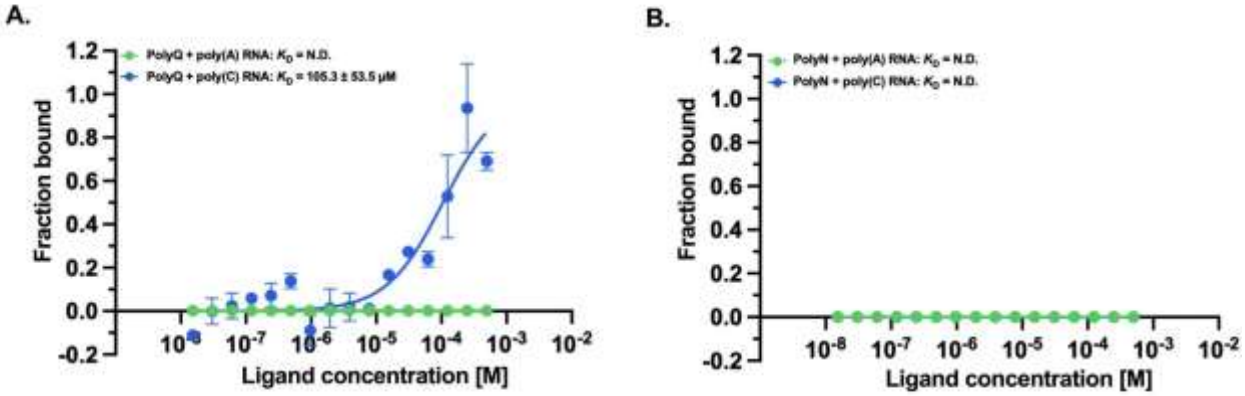

Figure S14. Thermodynamic plots of polyQ/N peptide with polyRNAs. Microscale thermophoresis was employed to determine the dissociation constants of polyQ/N peptide (6-mer) with poly(A)/(C) RNA. **(A)** polyQ peptide and **(B)** polyN peptide. The error bar represents the mean ± SE from three independent experimental replicates.

References

(1) Nishigaki, K.; Taguchi, K.; Kinoshita, Y.; Aita, T.; Husimi, Y. Y-ligation: An efficient method for ligating single-stranded DNAs and RNAs with T4 RNA ligase. *Mol. Divers.* **1998**, *4* (3), 187–190.

(2) Reyes, S. G.; Kuruma, Y.; Fujimi, M.; Yamazaki, M.; Eto, S.; Nishikawa, S.; Tamaki, S.; Kobayashi, A.; Mizuuchi, R.; Rothschild, L.; Ditzler, M.; Fujishima, K. PURE mRNA display and cDNA display provide rapid detection of core epitope motif *via* high-throughput sequencing. *Biotechnol. Bioeng.* **2021**, *118* (4), 1736–1749.

(3) Alam, K. K.; Chang, J. L.; Burke, D. H. FASTAptamer: A bioinformatic toolkit for high-throughput sequence analysis of combinatorial selections. *Mol. Ther.- Nucleic Acids* **2015**, *4* (3), e230.

(4) Crooks, G. E.; Hon, G.; Chandonia, J.-M.; Brenner, S. E. WebLogo: A sequence logo generator. *Genome Research* **2004**, *14* (6), 1188–1190.

(5) Osorio, D.; Rondón-Villarreal, P.; Torres, R. Peptides: A package for data mining of antimicrobial peptides. *Small* **2015**, *12*, 44–444.

(6) Kyte, J.; Doolittle, R. F. A simple method for displaying the hydropathic character of a protein. *J. Mol. Biol.* **1982**, *157* (1), 105–132.

(7) Butterworth, P. J. Lehninger: Principles of biochemistry (4th Edn) D. L. Nelson and M. C. Cox, W. H. Freeman & Co., New York, 1119 Pp (plus 17 Pp Glossary), ISBN 0-7167-4339-6 (2004). *Cell Biochem. Funct.* **2005**, *23* (4), 293–294.

(8) Sato, K.; Akiyama, M.; Sakakibara, Y. RNA secondary structure prediction using deep learning with thermodynamic integration. *Nat. Commun.* **2021**, *12* (1), 941.
